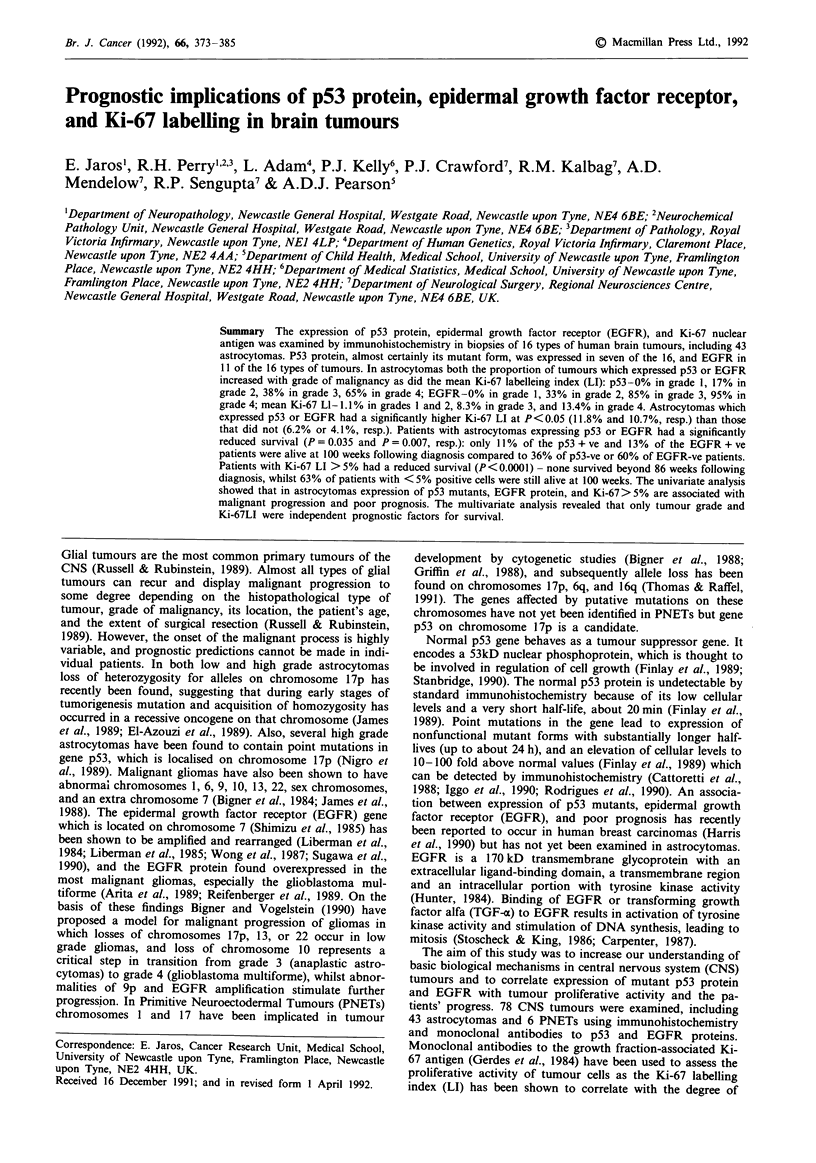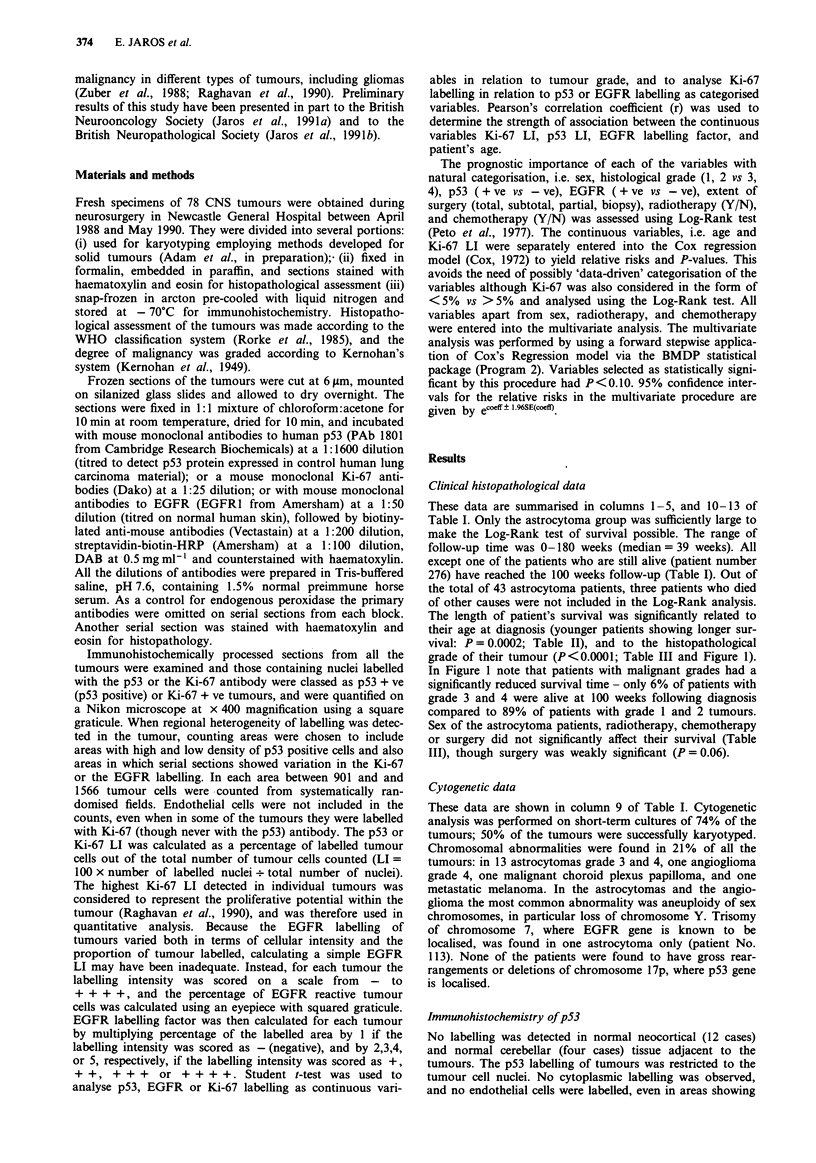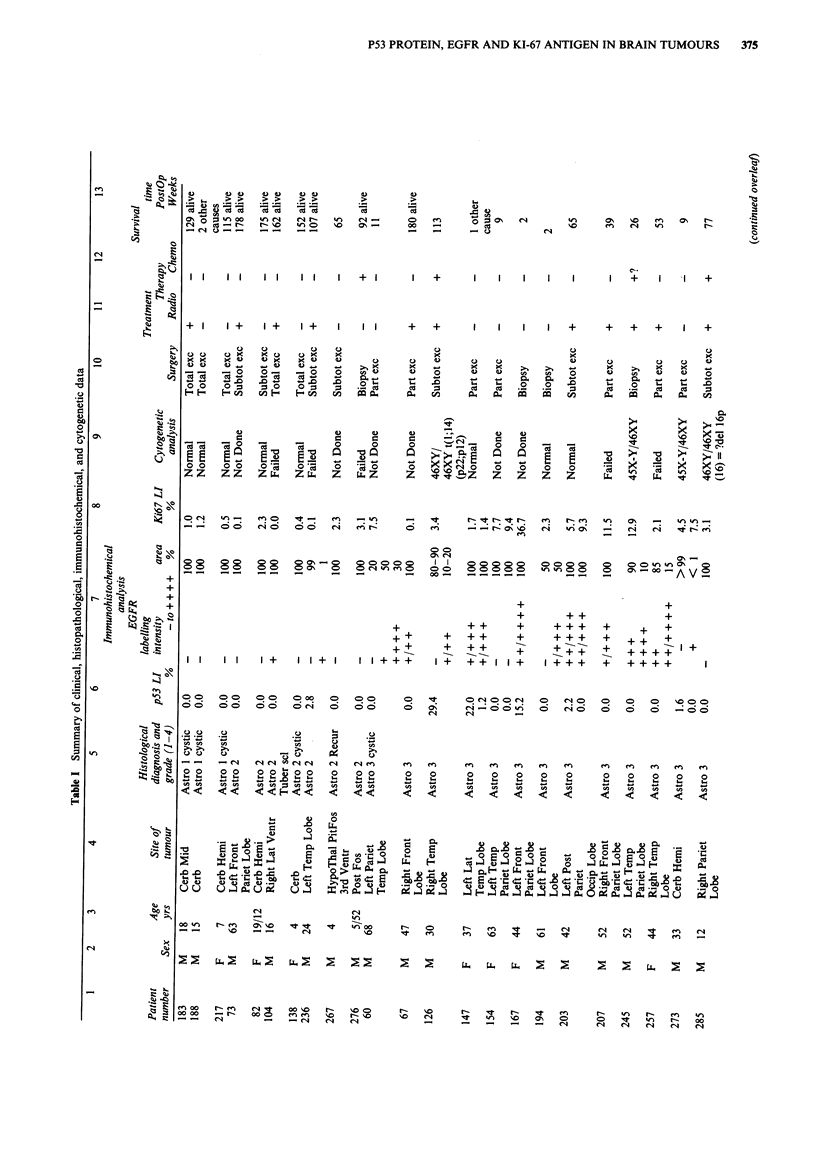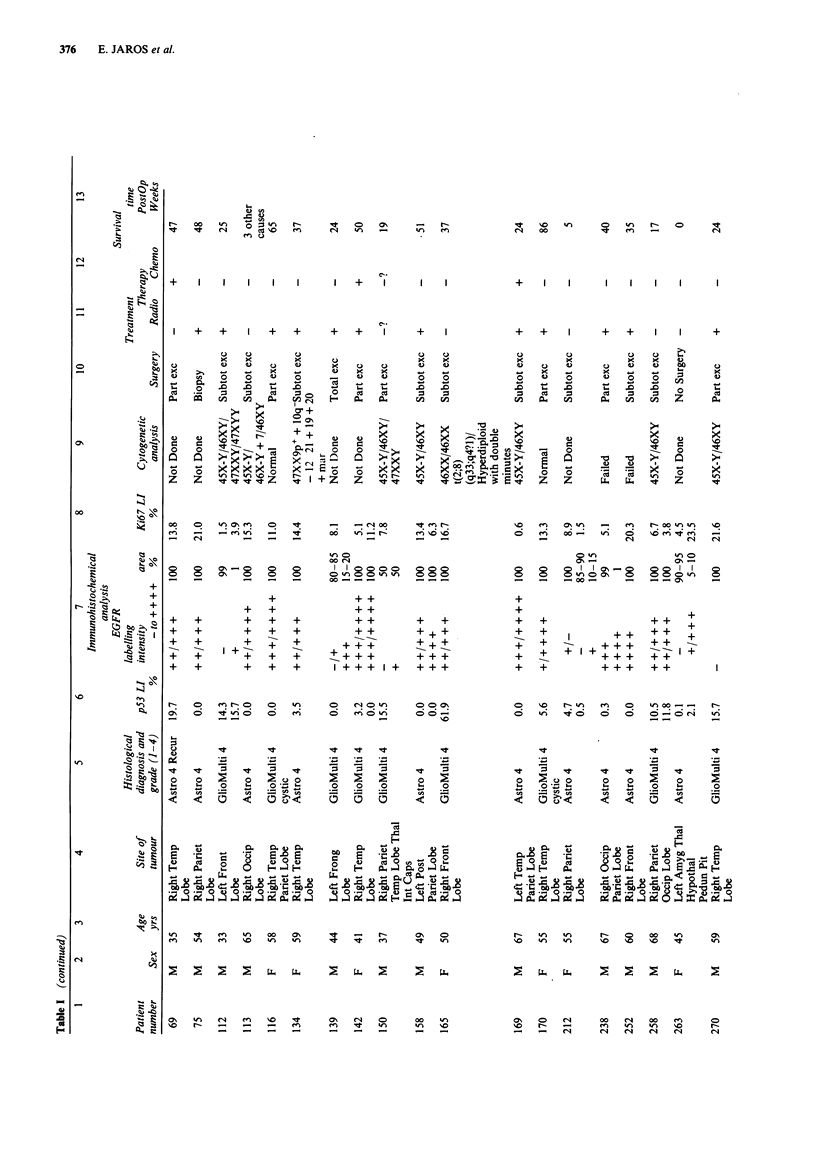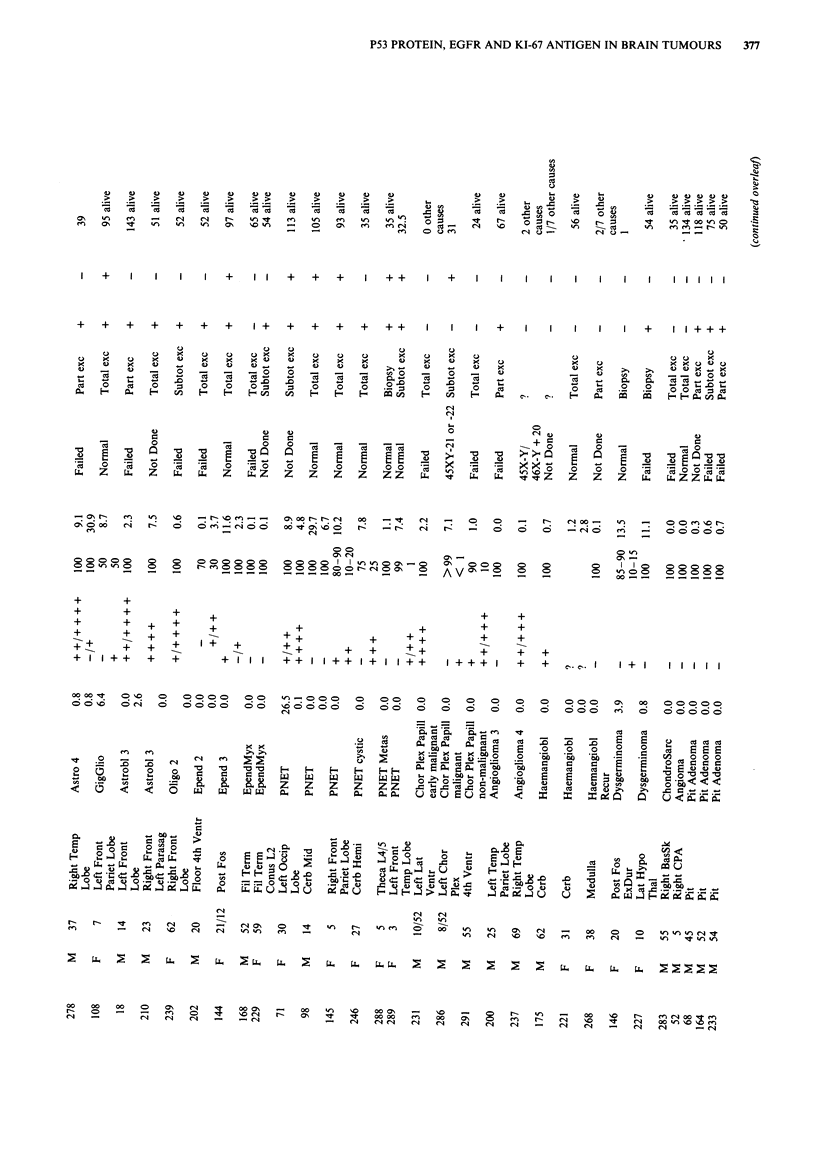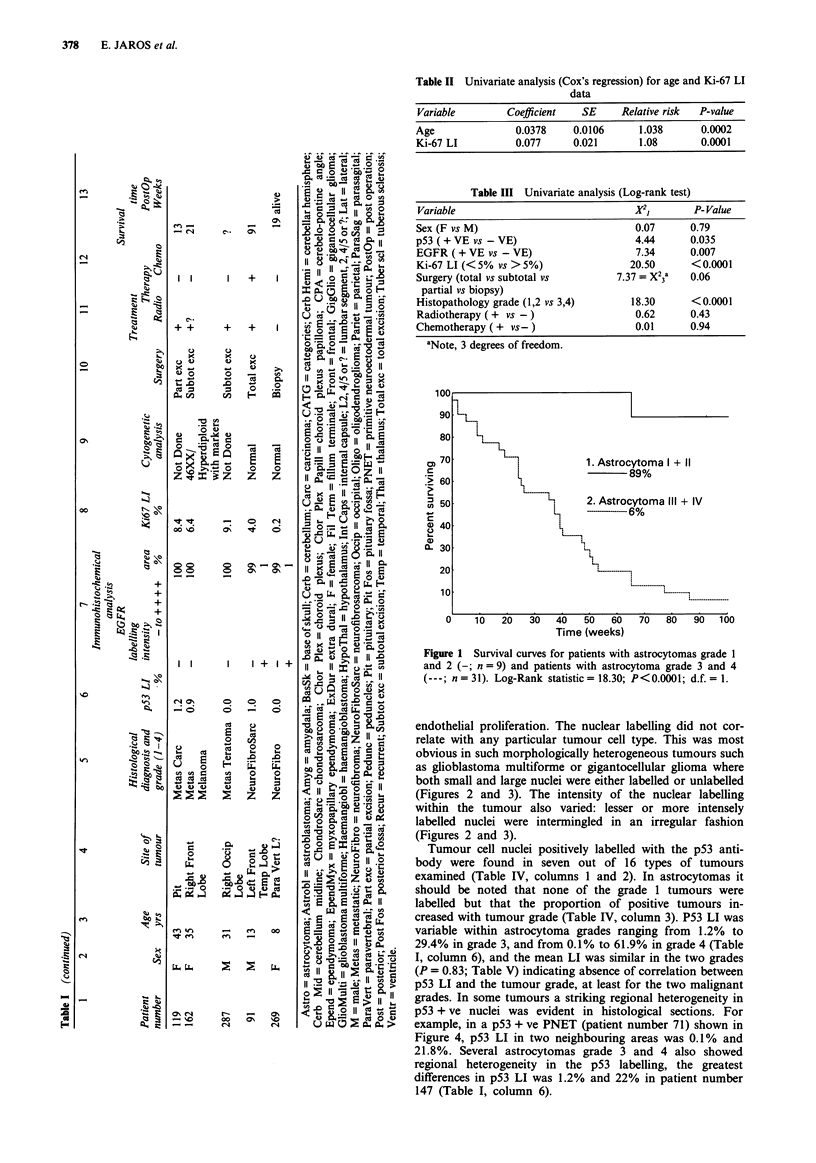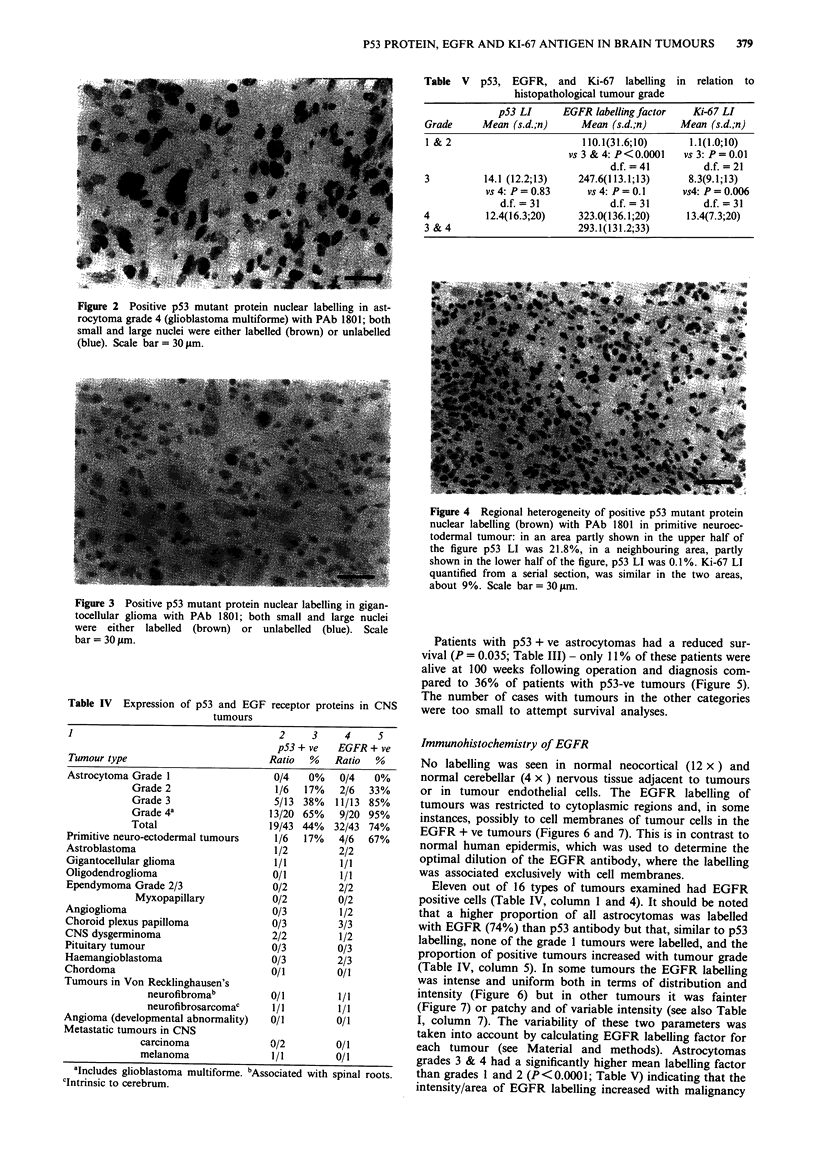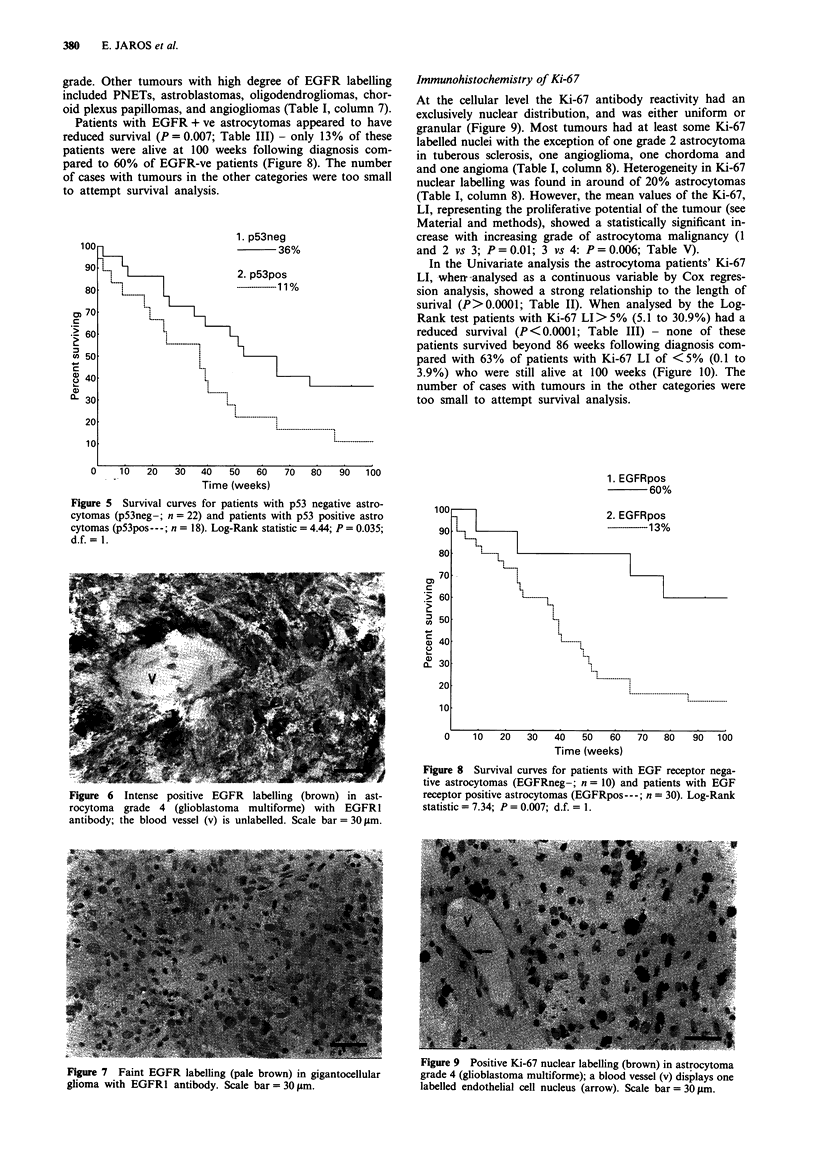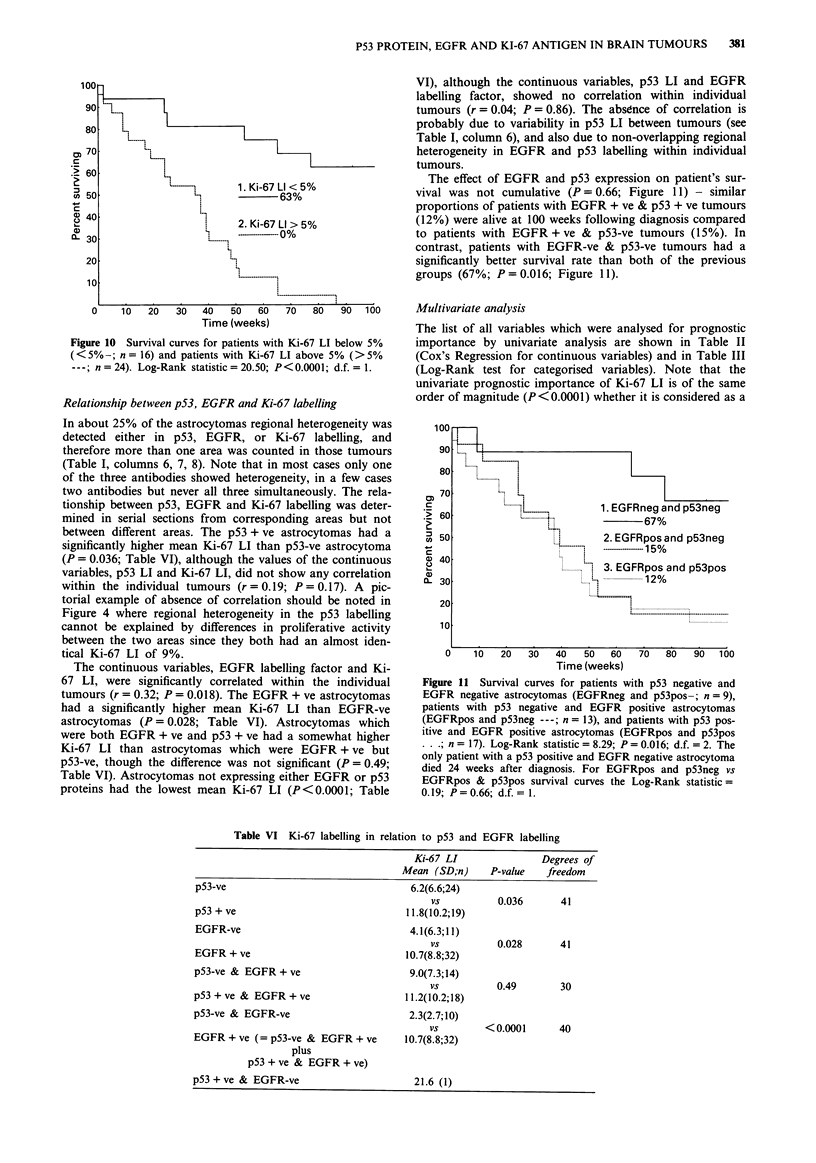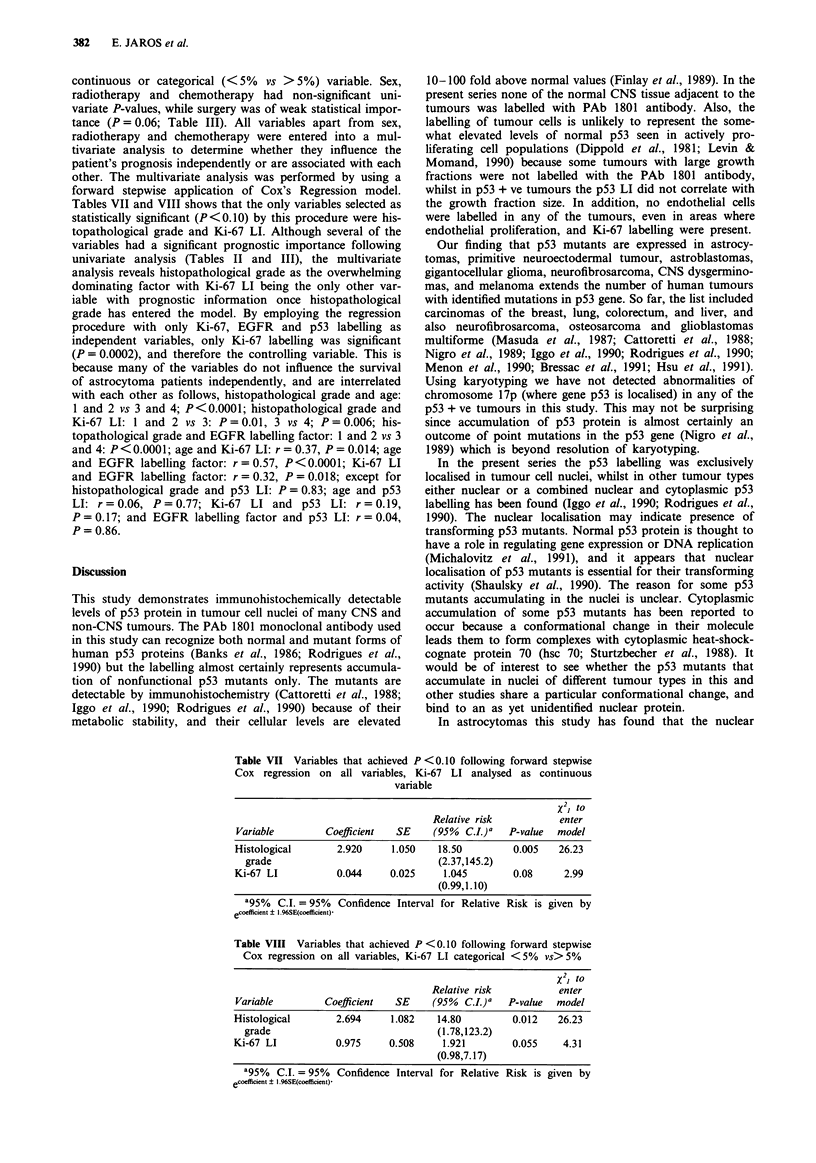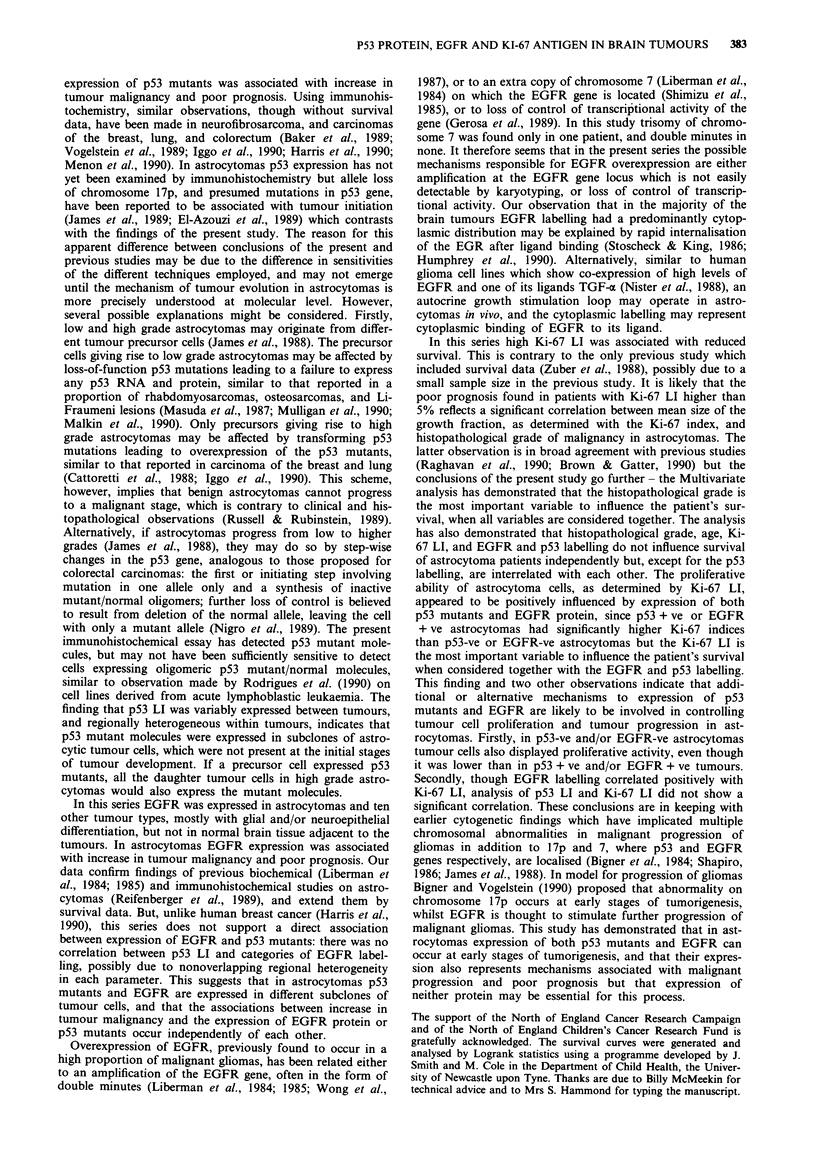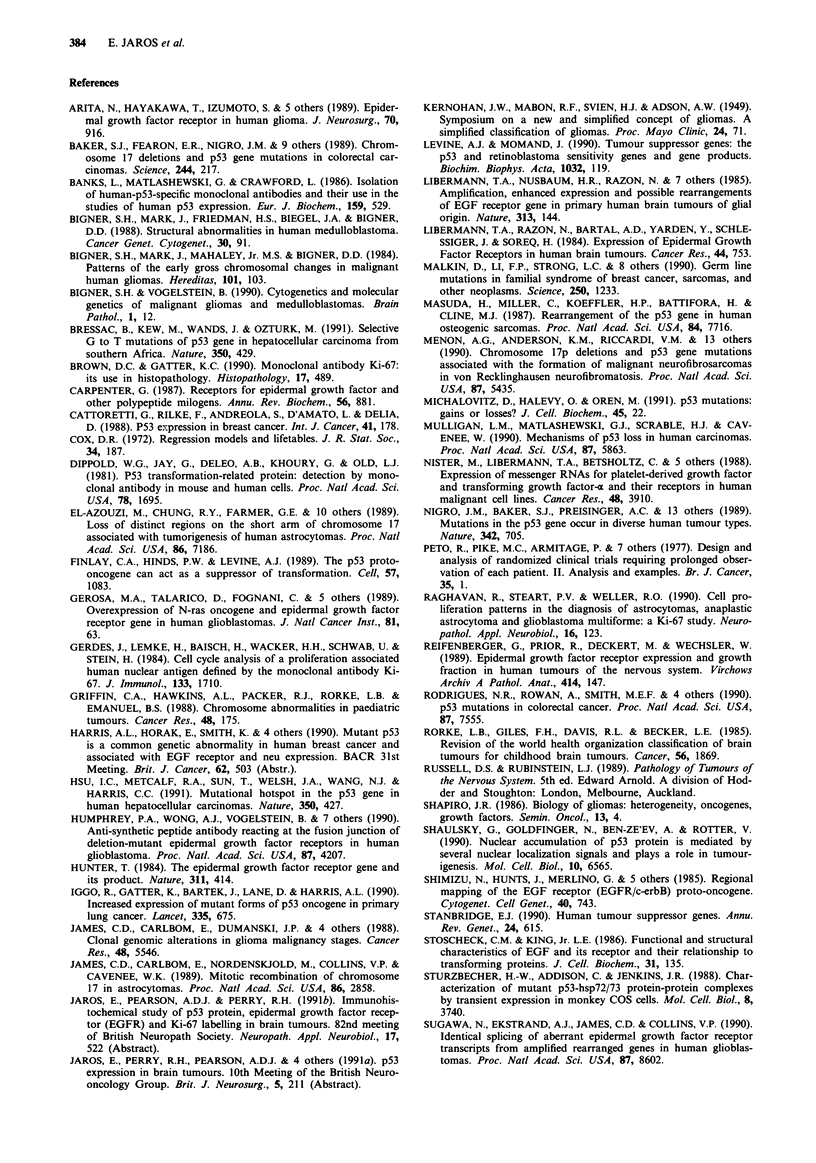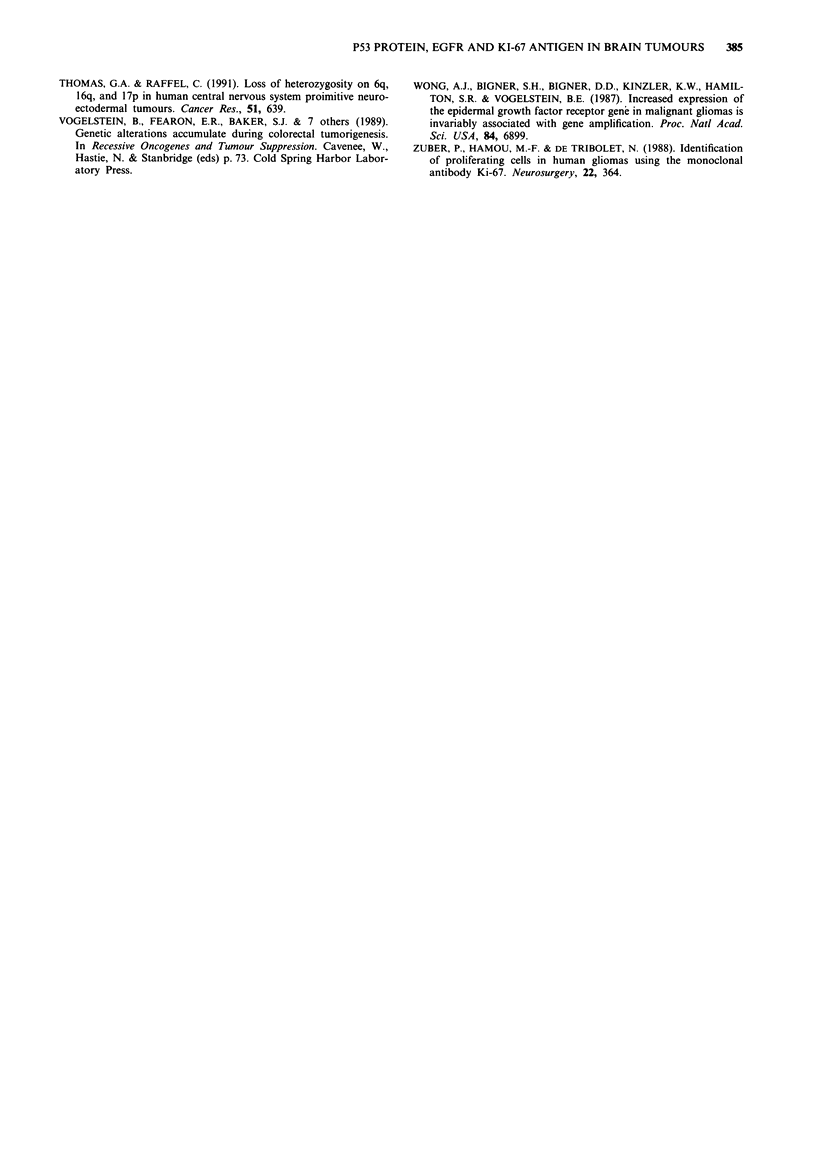# Prognostic implications of p53 protein, epidermal growth factor receptor, and Ki-67 labelling in brain tumours.

**DOI:** 10.1038/bjc.1992.273

**Published:** 1992-08

**Authors:** E. Jaros, R. H. Perry, L. Adam, P. J. Kelly, P. J. Crawford, R. M. Kalbag, A. D. Mendelow, R. P. Sengupta, A. D. Pearson

**Affiliations:** Department of Neuropathology, Newcastle General Hospital, Newcastle upon Tyne, UK.

## Abstract

**Images:**


Br. J. Cancer (1992), 66, 373-385 © Macmillan Press Ltd., 1992

### Prognostic implications of p53 protein, epidermal growth factor receptor, and Ki-67 labelling in brain tumours

E. Jaros[1], R.H. Perry[1,2,3], L. Adam[4], P.J. Kelly[6], P.J. Crawford[7], R.M. Kalbag[7], A.D. Mendelow[7], R.P. Sengupta[7] & A.D.J. Pearson[5]

[1]*Department of Neuropathology, Newcastle General Hospital, Westgate Road, Newcastle upon Tyne, NE4 6BE;* [2]*Neurochemical Pathology Unit, Newcastle General Hospital, Westgate Road, Newcastle upon Tyne, NE4 6BE;* [3]*Department of Pathology, Royal Victoria Infirmary, Newcastle upon Tyne, NE1 4LP;* [4]*Department of Human Genetics, Royal Victoria Infirmary, Claremont Place, Newcastle upon Tyne, NE2 4AA;* [5]*Department of Child Health, Medical School, University of Newcastle upon Tyne, Framlington Place, Newcastle upon Tyne, NE2 4HH;* [6]*Department of Medical Statistics, Medical School, University of Newcastle upon Tyne, Framlington Place, Newcastle upon Tyne, NE2 4HH;* [7]*Department of Neurological Surgery, Regional Neurosciences Centre, Newcastle General Hospital, Westgate Road, Newcastle upon Tyne, NE4 6BE, UK.*

**Summary** The expression of p53 protein, epidermal growth factor receptor (EGFR), and Ki-67 nuclear antigen was examined by immunohistochemistry in biopsies of 16 types of human brain tumours, including 43 astrocytomas. P53 protein, almost certainly its mutant form, was expressed in seven of the 16, and EGFR in 11 of the 16 types of tumours. In astrocytomas both the proportion of tumours which expressed p53 or EGFR increased with grade of malignancy as did the mean Ki-67 labelleing index (LI): p53-0% in grade 1, 17% in grade 2, 38% in grade 3, 65% in grade 4; EGFR-0% in grade 1, 33% in grade 2, 85% in grade 3, 95% in grade 4; mean Ki-67 Ll-1.1% in grades 1 and 2, 8.3% in grade 3, and 13.4% in grade 4. Astrocytomas which expressed p53 or EGFR had a significantly higher Ki-67 LI at $P<0.05$ (11.8% and 10.7%, resp.) than those that did not (6.2% or 4.1%, resp.). Patients with astrocytomas expressing p53 or EGFR had a significantly reduced survival ($P = 0.035$ and $P = 0.007$, resp.): only 11% of the p53 + ve and 13% of the EGFR + ve patients were alive at 100 weeks following diagnosis compared to 36% of p53-ve or 60% of EGFR-ve patients. Patients with Ki-67 LI >5% had a reduced survival ($P<0.0001$) - none survived beyond 86 weeks following diagnosis, whilst 63% of patients with <5% positive cells were still alive at 100 weeks. The univariate analysis showed that in astrocytomas expression of p53 mutants, EGFR protein, and Ki-67>5% are associated with malignant progression and poor prognosis. The multivariate analysis revealed that only tumour grade and Ki-67LI were independent prognostic factors for survival.

Glial tumours are the most common primary tumours of the CNS (Russell & Rubinstein, 1989). Almost all types of glial tumours can recur and display malignant progression to some degree depending on the histopathological type of tumour, grade of malignancy, its location, the patient's age, and the extent of surgical resection (Russell & Rubinstein, 1989). However, the onset of the malignant process is highly variable, and prognostic predictions cannot be made in individual patients. In both low and high grade astrocytomas loss of heterozygosity for alleles on chromosome 17p has recently been found, suggesting that during early stages of tumorigenesis mutation and acquisition of homozygosity has occurred in a recessive oncogene on that chromosome (James *et al.*, 1989; El-Azouzi *et al.*, 1989). Also, several high grade astrocytomas have been found to contain point mutations in gene p53, which is localised on chromosome 17p (Nigro *et al.*, 1989). Malignant gliomas have also been shown to have abnormai chromosomes 1, 6, 9, 10, 13, 22, sex chromosomes, and an extra chromosome 7 (Bigner *et al.*, 1984; James *et al.*, 1988). The epidermal growth factor receptor (EGFR) gene which is located on chromosome 7 (Shimizu *et al.*, 1985) has been shown to be amplified and rearranged (Liberman *et al.*, 1984; Liberman *et al.*, 1985; Wong *et al.*, 1987; Sugawa *et al.*, 1990), and the EGFR protein found overexpressed in the most malignant gliomas, especially the glioblastoma multiforme (Arita *et al.*, 1989; Reifenberger *et al.*, 1989. On the basis of these findings Bigner and Vogelstein (1990) have proposed a model for malignant progression of gliomas in which losses of chromosomes 17p, 13, or 22 occur in low grade gliomas, and loss of chromosome 10 represents a critical step in transition from grade 3 (anaplastic astrocytomas) to grade 4 (glioblastoma multiforme), whilst abnormalities of 9p and EGFR amplification stimulate further progression. In Primitive Neuroectodermal Tumours (PNETs) chromosomes 1 and 17 have been implicated in tumour development by cytogenetic studies (Bigner *et al.*, 1988; Griffin *et al.*, 1988), and subsequently allele loss has been found on chromosomes 17p, 6q, and 16q (Thomas & Raffel, 1991). The genes affected by putative mutations on these chromosomes have not yet been identified in PNETs but gene p53 on chromosome 17p is a candidate.

Normal p53 gene behaves as a tumour suppressor gene. It encodes a 53kD nuclear phosphoprotein, which is thought to be involved in regulation of cell growth (Finlay *et al.*, 1989; Stanbridge, 1990). The normal p53 protein is undetectable by standard immunohistochemistry because of its low cellular levels and a very short half-life, about 20 min (Finlay *et al.*, 1989). Point mutations in the gene lead to expression of nonfunctional mutant forms with substantially longer half-lives (up to about 24 h), and an elevation of cellular levels to 10-100 fold above normal values (Finlay *et al.*, 1989) which can be detected by immunohistochemistry (Cattoretti *et al.*, 1988; Iggo *et al.*, 1990; Rodrigues *et al.*, 1990). An association between expression of p53 mutants, epidermal growth factor receptor (EGFR), and poor prognosis has recently been reported to occur in human breast carcinomas (Harris *et al.*, 1990) but has not yet been examined in astrocytomas. EGFR is a 170 kD transmembrane glycoprotein with an extracellular ligand-binding domain, a transmembrane region and an intracellular portion with tyrosine kinase activity (Hunter, 1984). Binding of EGFR or transforming growth factor alfa (TGF-$\alpha$) to EGFR results in activation of tyrosine kinase activity and stimulation of DNA synthesis, leading to mitosis (Stoscheck & King, 1986; Carpenter, 1987).

The aim of this study was to increase our understanding of basic biological mechanisms in central nervous system (CNS) tumours and to correlate expression of mutant p53 protein and EGFR with tumour proliferative activity and the patients' progress. 78 CNS tumours were examined, including 43 astrocytomas and 6 PNETs using immunohistochemistry and monoclonal antibodies to p53 and EGFR proteins. Monoclonal antibodies to the growth fraction-associated Ki-67 antigen (Gerdes *et al.*, 1984) have been used to assess the proliferative activity of tumour cells as the Ki-67 labelling index (LI) has been shown to correlate with the degree of

Correspondence: E. Jaros, Cancer Research Unit, Medical School, University of Newcastle upon Tyne, Framlington Place, Newcastle upon Tyne, NE2 4HH, UK.
Received 16 December 1991; and in revised form 1 April 1992.

malignancy in different types of tumours, including gliomas (Zuber et al., 1988; Raghavan et al., 1990). Preliminary results of this study have been presented in part to the British Neurooncology Society (Jaros et al., 1991a) and to the British Neuropathological Society (Jaros et al., 1991b).

### Materials and methods

Fresh specimens of 78 CNS tumours were obtained during neurosurgery in Newcastle General Hospital between April 1988 and May 1990. They were divided into several portions: (i) used for karyotyping employing methods developed for solid tumours (Adam et al., in preparation); (ii) fixed in formalin, embedded in paraffin, and sections stained with haematoxylin and eosin for histopathological assessment (iii) snap-frozen in arcton pre-cooled with liquid nitrogen and stored at − 70°C for immunohistochemistry. Histopathological assessment of the tumours was made according to the WHO classification system (Rorke et al., 1985), and the degree of malignancy was graded according to Kernohan's system (Kernohan et al., 1949).

Frozen sections of the tumours were cut at 6 μm, mounted on silanized glass slides and allowed to dry overnight. The sections were fixed in 1:1 mixture of chloroform:acetone for 10 min at room temperature, dried for 10 min, and incubated with mouse monoclonal antibodies to human p53 (PAb 1801 from Cambridge Research Biochemicals) at a 1:1600 dilution (titred to detect p53 protein expressed in control human lung carcinoma material); or a mouse monoclonal Ki-67 antibodies (Dako) at a 1:25 dilution; or with mouse monoclonal antibodies to EGFR (EGFR1 from Amersham) at a 1:50 dilution (titred on normal human skin), followed by biotinylated anti-mouse antibodies (Vectastain) at a 1:200 dilution, streptavidin-biotin-HRP (Amersham) at a 1:100 dilution, DAB at 0.5 mg $ml^{-1}$ and counterstained with haematoxylin. All the dilutions of antibodies were prepared in Tris-buffered saline, pH 7.6, containing 1.5% normal preimmune horse serum. As a control for endogenous peroxidase the primary antibodies were omitted on serial sections from each block. Another serial section was stained with haematoxylin and eosin for histopathology.

Immunohistochemically processed sections from all the tumours were examined and those containing nuclei labelled with the p53 or the Ki-67 antibody were classed as p53 + ve (p53 positive) or Ki-67 + ve tumours, and were quantified on a Nikon microscope at × 400 magnification using a square graticule. When regional heterogeneity of labelling was detected in the tumour, counting areas were chosen to include areas with high and low density of p53 positive cells and also areas in which serial sections showed variation in the Ki-67 or the EGFR labelling. In each area between 901 and and 1566 tumour cells were counted from systematically randomised fields. Endothelial cells were not included in the counts, even when in some of the tumours they were labelled with Ki-67 (though never with the p53) antibody. The p53 or Ki-67 LI was calculated as a percentage of labelled tumour cells out of the total number of tumour cells counted (LI = 100 × number of labelled nuclei ÷ total number of nuclei). The highest Ki-67 LI detected in individual tumours was considered to represent the proliferative potential within the tumour (Raghavan et al., 1990), and was therefore used in quantitative analysis. Because the EGFR labelling of tumours varied both in terms of cellular intensity and the proportion of tumour labelled, calculating a simple EGFR LI may have been inadequate. Instead, for each tumour the labelling intensity was scored on a scale from − to + + + +, and the percentage of EGFR reactive tumour cells was calculated using an eyepiece with squared graticule. EGFR labelling factor was then calculated for each tumour by multiplying percentage of the labelled area by 1 if the labelling intensity was scored as − (negative), and by 2,3,4, or 5, respectively, if the labelling intensity was scored as +, + +, + + + or + + + +. Student t-test was used to analyse p53, EGFR or Ki-67 labelling as continuous variables in relation to tumour grade, and to analyse Ki-67 labelling in relation to p53 or EGFR labelling as categorised variables. Pearson's correlation coefficient (r) was used to determine the strength of association between the continuous variables Ki-67 LI, p53 LI, EGFR labelling factor, and patient's age.

The prognostic importance of each of the variables with natural categorisation, i.e. sex, histological grade (1, 2 vs 3, 4), p53 ( + ve vs − ve), EGFR ( + ve vs − ve), extent of surgery (total, subtotal, partial, biopsy), radiotherapy (Y/N), and chemotherapy (Y/N) was assessed using Log-Rank test (Peto et al., 1977). The continuous variables, i.e. age and Ki-67 LI were separately entered into the Cox regression model (Cox, 1972) to yield relative risks and P-values. This avoids the need of possibly 'data-driven' categorisation of the variables although Ki-67 was also considered in the form of $<5\%$ vs $>5\%$ and analysed using the Log-Rank test. All variables apart from sex, radiotherapy, and chemotherapy were entered into the multivariate analysis. The multivariate analysis was performed by using a forward stepwise application of Cox's Regression model via the BMDP statistical package (Program 2). Variables selected as statistically significant by this procedure had $P<0.10$. 95% confidence intervals for the relative risks in the multivariate procedure are given by $e^{coeff \pm 1.96SE(coeff)}$.

### Results

### Clinical histopathological data

These data are summarised in columns 1–5, and 10–13 of Table I. Only the astrocytoma group was sufficiently large to make the Log-Rank test of survival possible. The range of follow-up time was 0–180 weeks (median = 39 weeks). All except one of the patients who are still alive (patient number 276) have reached the 100 weeks follow-up (Table I). Out of the total of 43 astrocytoma patients, three patients who died of other causes were not included in the Log-Rank analysis. The length of patient's survival was significantly related to their age at diagnosis (younger patients showing longer survival: $P = 0.0002$; Table II), and to the histopathological grade of their tumour ($P<0.0001$; Table III and Figure 1). In Figure 1 note that patients with malignant grades had a significantly reduced survival time – only 6% of patients with grade 3 and 4 were alive at 100 weeks following diagnosis compared to 89% of patients with grade 1 and 2 tumours. Sex of the astrocytoma patients, radiotherapy, chemotherapy or surgery did not significantly affect their survival (Table III), though surgery was weakly significant ($P = 0.06$).

### Cytogenetic data

These data are shown in column 9 of Table I. Cytogenetic analysis was performed on short-term cultures of 74% of the tumours; 50% of the tumours were successfully karyotyped. Chromosomal abnormalities were found in 21% of all the tumours: in 13 astrocytomas grade 3 and 4, one angioglioma grade 4, one malignant choroid plexus papilloma, and one metastatic melanoma. In the astrocytomas and the angioglioma the most common abnormality was aneuploidy of sex chromosomes, in particular loss of chromosome Y. Trisomy of chromosome 7, where EGFR gene is known to be localised, was found in one astrocytoma only (patient No. 113). None of the patients were found to have gross rearrangements or deletions of chromosome 17p, where p53 gene is localised.

### Immunohistochemistry of p53

No labelling was detected in normal neocortical (12 cases) and normal cerebellar (four cases) tissue adjacent to the tumours. The p53 labelling of tumours was restricted to the tumour cell nuclei. No cytoplasmic labelling was observed, and no endothelial cells were labelled, even in areas showing

**Table I** Summary of clinical, histopathological, immunohistochemical, and cytogenetic data

| 1 | 2 | 3 | 4 | 5 | 6 | 7 | | 8 | 9 | 10 | 11 | 12 | 13 |
|---|---|---|---|---|---|---|---|---|---|---|---|---|---|
| | | | | | | Immunohistochemical analysis | | | | | Treatment | | |
| | | | | | | EGFR | | | | | Therapy | | |
| Patient number | Sex | Age yrs | Site of tumour | Histological diagnosis and grade (1–4) | p53 LI % | labelling intensity – to ++++ | area % | Ki67 LI % | Cytogenetic analysis | Surgery | Radio | Chemo | Survival time PostOp Weeks |
| 183 | M | 18 | Cerb Mid | Astro 1 cystic | 0.0 | – | 100 | 1.0 | Normal | Total exc | + | – | 129 alive |
| 188 | M | 15 | Cerb | Astro 1 cystic | 0.0 | – | 100 | 1.2 | Normal | Total exc | – | – | 2 other causes |
| 217 | F | 7 | Cerb Hemi | Astro 1 cystic | 0.0 | – | 100 | 0.5 | Normal | Total exc | – | – | 115 alive |
| 73 | M | 63 | Left Front Pariet Lobe | Astro 2 | 0.0 | – | 100 | 0.1 | Not Done | Subtot exc | + | – | 178 alive |
| 82 | F | 19/12 | Cerb Hemi | Astro 2 | 0.0 | – | 100 | 2.3 | Normal | Subtot exc | – | – | 175 alive |
| 104 | M | 16 | Right Lat Ventr | Astro 2 Tuber scl | 0.0 | + | 100 | 0.0 | Failed | Total exc | + | – | 162 alive |
| 138 | F | 4 | Cerb | Astro 2 cystic | 0.0 | – | 100 | 0.4 | Normal | Total exc | – | – | 152 alive |
| 236 | M | 24 | Left Temp Lobe | Astro 2 | 2.8 | –<br>+ | 99<br>1 | 0.1 | Failed | Subtot exc | + | – | 107 alive |
| 267 | M | 4 | HypoThal PitFos 3rd Ventr | Astro 2 Recur | 0.0 | – | 100 | 2.3 | Not Done | Subtot exc | – | – | 65 |
| 276 | M | 5/52 | Post Fos | Astro 2 | 0.0 | – | 100 | 3.1 | Failed | Biopsy | – | + | 92 alive |
| 60 | M | 68 | Left Pariet Temp Lobe | Astro 3 cystic | 0.0 | –<br>+<br>++++ | 20<br>50<br>30 | 7.5 | Not Done | Part exc | – | – | 11 |
| 67 | M | 47 | Right Front Lobe | Astro 3 | 0.0 | +/++ | 100 | 0.1 | Not Done | Part exc | + | – | 180 alive |
| 126 | M | 30 | Right Temp Lobe | Astro 3 | 29.4 | –<br>+/++ | 80–90<br>10–20 | 3.4 | 46XY/ 46XY t(1;14) (p22;p12) | Subtot exc | + | + | 113 |
| 147 | F | 37 | Left Lat Temp Lobe | Astro 3 | 22.0<br>1.2 | +/+++<br>+/+++ | 100<br>100 | 1.7<br>1.4 | Normal | Part exc | – | – | 1 other cause |
| 154 | F | 63 | Left Temp Pariet Lobe | Astro 3 | 0.0<br>0.0 | –<br>– | 100<br>100 | 7.7<br>9.4 | Not Done | Part exc | – | – | 9 |
| 167 | F | 44 | Left Front Pariet Lobe | Astro 3 | 15.2 | ++/++++ | 100 | 36.7 | Not Done | Biopsy | – | – | 2 |
| 194 | M | 61 | Left Front Lobe | Astro 3 | 0.0 | –<br>+/+++ | 50<br>50 | 2.3 | Normal | Biopsy | – | – | 2 |
| 203 | M | 42 | Left Post Pariet Occip Lobe | Astro 3 | 2.2<br>0.0 | ++/+++<br>++/+++ | 100<br>100 | 5.7<br>9.3 | Normal | Subtot exc | + | – | 65 |
| 207 | M | 52 | Right Front Pariet Lobe | Astro 3 | 0.0 | +/+++ | 100 | 11.5 | Failed | Part exc | + | – | 39 |
| 245 | M | 52 | Left Temp Pariet Lobe | Astro 3 | 0.0 | +++<br>++++ | 90<br>10 | 12.9 | 45X-Y/46XY | Biopsy | + | +? | 26 |
| 257 | F | 44 | Right Temp Lobe | Astro 3 | 0.0 | ++<br>++/++++ | 85<br>15 | 2.1 | Failed | Part exc | + | – | 53 |
| 273 | M | 33 | Cerb Hemi | Astro 3 | 1.6<br>0.0 | –<br>+ | >99<br>< 1 | 4.5<br>7.5 | 45X-Y/46XY | Part exc | – | – | 9 |
| 285 | M | 12 | Right Pariet Lobe | Astro 3 | 0.0 | – | 100 | 3.1 | 46XY/46XY (16) = ?del 16p | Subtot exc | + | + | 77 |

(continued overleaf)

**Table I** *(continued)*

| 1 | 2 | 3 | 4 | 5 | 6 | 7 | | 8 | 9 | 10 | 11 | 12 | 13 |
|---|---|---|---|---|---|---|---|---|---|---|---|---|---|
| | | | | | | *Immunohistochemical analysis EGFR* | | | | | *Treatment* | | *Survival* |
| *Patient number* | *Sex* | *Age yrs* | *Site of tumour* | *Histological diagnosis and grade (1–4)* | *p53 LI %* | *labelling intensity – to ++++* | *area %* | *Ki67 LI %* | *Cytogenetic analysis* | *Surgery* | *Therapy Radio* | *Therapy Chemo* | *time PostOp Weeks* |
| 69 | M | 35 | Right Temp Lobe | Astro 4 Recur | 19.7 | ++/+++ | 100 | 13.8 | Not Done | Part exc | – | + | 47 |
| 75 | M | 54 | Right Pariet Lobe | Astro 4 | 0.0 | ++/+++ | 100 | 21.0 | Not Done | Biopsy | + | – | 48 |
| 112 | M | 33 | Left Front Lobe | GlioMulti 4 | 14.3<br>15.7 | –<br>+ | 99<br>1 | 1.5<br>3.9 | 45X-Y/46XY/ 47XXY/47XYY | Subtot exc | + | – | 25 |
| 113 | M | 65 | Right Occip Lobe | Astro 4 | 0.0 | ++/++++ | 100 | 15.3 | 45X-Y/ 46X-Y + 7/46XY | Subtot exc | – | – | 3 other causes |
| 116 | F | 58 | Right Temp Pariet Lobe | GlioMulti 4 cystic | 0.0 | +++/++++ | 100 | 11.0 | Normal | Part exc | + | – | 65 |
| 134 | F | 59 | Right Temp Lobe | Astro 4 | 3.5 | ++/+++ | 100 | 14.4 | 47XX9p$^+$ + 10q$^-$ – 12 21 + 19 + 20 + mar | Subtot exc | + | – | 37 |
| 139 | M | 44 | Left Frong Lobe | GlioMulti 4 | 0.0 | –/+<br>+++ | 80–85<br>15–20 | 8.1 | Not Done | Total exc | + | – | 24 |
| 142 | F | 41 | Right Temp Lobe | GlioMulti 4 | 3.2<br>0.0 | +++/++++<br>+++/++++ | 100<br>100 | 5.1<br>11.2 | Not Done | Part exc | + | + | 50 |
| 150 | M | 37 | Right Pariet Temp Lobe Thal Int Caps | GlioMulti 4 | 15.5 | –<br>+ | 50<br>50 | 7.8 | 45X-Y/46XY/ 47XXY | Part exc | –? | –? | 19 |
| 158 | M | 49 | Left Post Pariet Lobe | Astro 4 | 0.0<br>0.0 | ++/+++<br>++++ | 100<br>100 | 13.4<br>6.3 | 45X-Y/46XY | Subtot exc | + | – | ·51 |
| 165 | F | 50 | Right Front Lobe | GlioMulti 4 | 61.9 | ++/+++ | 100 | 16.7 | 46XX/46XX t(2;8) (q33;q4?1)/ Hyperdiploid with double minutes | Subtot exc | – | – | 37 |
| 169 | M | 67 | Left Temp Pariet Lobe | Astro 4 | 0.0 | +++/++++ | 100 | 0.6 | 45X-Y/46XY | Subtot exc | + | + | 24 |
| 170 | F | 55 | Right Temp Lobe | GlioMulti 4 cystic | 5.6 | +/++++ | 100 | 13.3 | Normal | Part exc | + | – | 86 |
| 212 | F | 55 | Right Pariet Lobe | Astro 4 | 4.7<br>0.5 | +/–<br>–<br>+ | 100<br>85–90<br>10–15 | 8.9<br>1.5 | Not Done | Subtot exc | – | – | 5 |
| 238 | M | 67 | Right Occip Pariet Lobe | Astro 4 | 0.3 | +++<br>++++ | 99<br>1 | 5.1 | Failed | Part exc | + | – | 40 |
| 252 | M | 60 | Right Front Lobe | Astro 4 | 0.0 | ++++ | 100 | 20.3 | Failed | Subtot exc | + | – | 35 |
| 258 | M | 68 | Right Pariet Occip Lobe | GlioMulti 4 | 10.5<br>11.8 | ++/+++<br>++/+++ | 100<br>100 | 6.7<br>3.8 | 45X-Y/46XY | Subtot exc | – | – | 17 |
| 263 | F | 45 | Left Amyg Thal Hypothal Pedun Pit | Astro 4 | 0.1<br>2.1 | –<br>+/+++ | 90–95<br>5–10 | 4.5<br>23.5 | Not Done | No Surgery | – | – | 0 |
| 270 | M | 59 | Right Temp Lobe | GlioMulti 4 | 15.7 | – | 100 | 21.6 | 45X-Y/46XY | Part exc | + | – | 24 |

| | | | | | | | | | | | | | |
|---|---|---|---|---|---|---|---|---|---|---|---|---|---|
| 278 | M | 37 | Right Temp Lobe | Astro 4 | 0.8<br>0.8 | ++/++++<br>−/+ | 100<br>100 | 9.1<br>30.9 | Failed | Part exc | + | − | 39 |
| 108 | F | 7 | Left Front Pariet Lobe | GigGlio | 6.4 | −<br>+ | 50<br>50 | 8.7 | Normal | Total exc | + | + | 95 alive |
| 18 | M | 14 | Left Front Lobe | Astrobl 3 | 0.0<br>2.6 | ++/++++ | 100 | 2.3 | Failed | Part exc | + | − | 143 alive |
| 210 | M | 23 | Right Front Left Parasag | Astrobl 3 | 0.0 | ++++ | 100 | 7.5 | Not Done | Total exc | + | − | 51 alive |
| 239 | F | 62 | Right Front Lobe | Oligo 2 | 0.0 | +/++++ | 100 | 0.6 | Failed | Subtot exc | + | − | 52 alive |
| 202 | M | 20 | Floor 4th Ventr | Epend 2 | 0.0<br>0.0 | −<br>+/++ | 70<br>30 | 0.1<br>3.7 | Failed | Total exc | + | − | 52 alive |
| 144 | F | 21/12 | Post Fos | Epend 3 | 0.0 | +<br>−/+ | 100<br>100 | 11.6<br>2.3 | Normal | Total exc | + | + | 97 alive |
| 168 | M | 52 | Fil Term | EpendMyx | 0.0 | − | 100 | 0.1 | Failed | Total exc | − | − | 65 alive |
| 229 | F | 59 | Fil Term Conus L2 | EpendMyx | 0.0 | − | 100 | 0.1 | Not Done | Subtot exc | + | − | 54 alive |
| 71 | F | 30 | Left Occip Lobe | PNET | 26.5<br>0.1 | +/++<br>++++ | 100<br>100 | 8.9<br>4.8 | Not Done | Subtot exc | + | + | 113 alive |
| 98 | M | 14 | Cerb Mid | PNET | 0.0<br>0.0 | −<br>− | 100<br>100 | 29.7<br>6.7 | Normal | Total exc | + | + | 105 alive |
| 145 | F | 5 | Right Front Pariet Lobe | PNET | 0.0 | +<br>++ | 80–90<br>10–20 | 10.2 | Normal | Total exc | + | + | 93 alive |
| 246 | F | 27 | Cerb Hemi | PNET cystic | 0.0 | −<br>+++ | 75<br>25 | 7.8 | Normal | Total exc | + | − | 35 alive |
| 288 | F | 5 | Theca L4/5 | PNET Metas | 0.0 | − | 100 | 1.1 | Normal | Biopsy | + | + | 35 alive |
| 289 | F | 3 | Left Front Temp Lobe | PNET | 0.0 | −<br>+/++ | 99<br>1 | 7.4 | Normal | Subtot exc | + | + | 32.5 |
| 231 | M | 10/52 | Left Lat Ventr | Chor Plex Papill early malignant | 0.0 | ++++ | 100 | 2.2 | Failed | Total exc | − | − | 0 other causes |
| 286 | M | 8/52 | Left Chor Plex | Chor Plex Papill malignant | 0.0 | −<br>+ | >99<br>< 1 | 7.1 | 45XY-21 or -22 | Subtot exc | − | + | 31 |
| 291 | M | 55 | 4th Ventr | Chor Plex Papill non-malignant | 0.0 | +<br>++/+++ | 90<br>10 | 1.0 | Failed | Total exc | − | − | 24 alive |
| 200 | M | 25 | Left Temp Pariet Lobe | Angioglioma 3 | 0.0 | − | 100 | 0.0 | Failed | Part exc | + | − | 67 alive |
| 237 | M | 69 | Right Temp Lobe | Angioglioma 4 | 0.0 | ++/+++ | 100 | 0.1 | 45X-Y/<br>46X-Y + 20 | ? | − | − | 2 other causes |
| 175 | M | 62 | Cerb | Haemangiobl | 0.0 | ++ | 100 | 0.7 | Not Done | ? | − | − | 1/7 other causes |
| 221 | F | 31 | Cerb | Haemangiobl | 0.0<br>0.0 | ?<br>? | | 1.2<br>2.8 | Normal | Total exc | − | − | 56 alive |
| 268 | F | 38 | Medulla | Haemangiobl Recur | 0.0 | − | 100 | 0.1 | Not Done | Part exc | − | − | 2/7 other causes |
| 146 | F | 20 | Post Fos ExDur | Dysgerminoma | 3.9 | −<br>+ | 85–90<br>10–15 | 13.5 | Normal | Biopsy | − | − | 1 |
| 227 | F | 10 | Lat Hypo Thal | Dysgerminoma | 0.8 | − | 100 | 11.1 | Failed | Biopsy | + | − | 54 alive |
| 283 | M | 55 | Right BasSk | ChondroSarc | 0.0 | − | 100 | 0.0 | Failed | Total exc | − | − | 35 alive |
| 52 | M | 5 | Right CPA | Angioma | 0.0 | − | 100 | 0.0 | Normal | Total exc | − | − | 134 alive |
| 68 | M | 45 | Pit | Pit Adenoma | 0.0 | − | 100 | 0.3 | Not Done | Part exc | + | − | 118 alive |
| 164 | M | 52 | Pit | Pit Adenoma | 0.0 | − | 100 | 0.6 | Failed | Subtot exc | + | − | 75 alive |
| 233 | M | 54 | Pit | Pit Adenoma | 0.0 | − | 100 | 0.7 | Failed | Part exc | + | − | 50 alive |

*(continued overleaf)*

**Table I** *(continued)*

| 1 | 2 | 3 | 4 | 5 | 6 | 7 | | 8 | 9 | 10 | 11 | 12 | 13 |
|---|---|---|---|---|---|---|---|---|---|---|---|---|---|
| | | | | | | *Immunohistochemical analysis EGFR* | | | | | *Treatment* | | *Survival* |
| *Patient number* | *Sex* | *Age yrs* | *Site of tumour* | *Histological diagnosis and grade (1–4)* | *p53 LI %* | *labelling intensity – to + + + +* | *area %* | *Ki67 LI %* | *Cytogenetic analysis* | *Surgery* | *Therapy Radio* | *Chemo* | *time PostOp Weeks* |
| 119 | F | 43 | Pit | Metas Carc | 1.2 | – | 100 | 8.4 | Not Done | Part exc | + | – | 13 |
| 162 | F | 35 | Right Front Lobe | Metas Melanoma | 0.9 | – | 100 | 6.4 | 46XX/ Hyperdiploid with markers | Subtot exc | +? | – | 21 |
| 287 | M | 31 | Right Occip Lobe | Metas Teratoma | 0.0 | – | 100 | 9.1 | Not Done | Subtot exc | + | – | ? |
| 91 | M | 13 | Left Front Temp Lobe | NeuroFibroSarc | 1.0 | – | 99 | 4.0 | Normal | Total exc | + | + | 91 |
| | | | | | | + | 1 | | | | | | |
| 269 | F | 8 | Para Vert L? | NeuroFibro | 0.0 | – | 99 | 0.2 | Normal | Biopsy | – | – | 19 alive |
| | | | | | | + | 1 | | | | | | |

Astro = astrocytoma; Astrobl = astroblastoma; Amyg = amygdala; BasSk = base of skull; Cerb = cerebellum; Carc = carcinoma; CATG = categories; Cerb Hemi = cerebellar hemisphere; Cerb Mid = cerebellum midline; ChondroSarc = chondrosarcoma; Chor Plex = choroid plexus; Chor Plex Papill = choroid plexus papilloma; CPA = cerebelo-pontine angle; Epend = ependymoma; EpendMyx = myxopapillary ependymoma; ExDur = extra dural; F = female; Fil Term = fillum terminale; Front = frontal; GigGlio = gigantocellular glioma; GlioMulti = glioblastoma multiforme; Haemangiobl = haemangioblastoma; HypoThal = hypothalamus; Int Caps = internal capsule; L2, 4/5 or ? = lumbar segment, 2, 4/5 or ?; Lat = lateral; M = male; Metas = metastatic; NeuroFibro = neurofibroma; NeuroFibroSarc = neurofibrosarcoma; Occip = occipital; Oligo = oligodendroglioma; Pariet = parietal; ParaSag = parasagital; ParaVert = paravertebral; Part exc = partial excision; Pedunc = peduncles; Pit = pituitary; Pit Fos = pituitary fossa; PNET = primitive neuroectodermal tumour; PostOp = post operation; Post = posterior; Post Fos = posterior fossa; Recur = recurrent; Subtot exc = subtotal excision; Temp = temporal; Thal = thalamus; Total exc = total excision; Tuber scl = tuberous sclerosis; Ventr = ventricle.

**Table II** Univariate analysis (Cox's regression) for age and Ki-67 LI data

| *Variable* | *Coefficient* | *SE* | *Relative risk* | *P-value* |
|---|---|---|---|---|
| Age | 0.0378 | 0.0106 | 1.038 | 0.0002 |
| Ki-67 LI | 0.077 | 0.021 | 1.08 | 0.0001 |

**Table III** Univariate analysis (Log-rank test)

| *Variable* | $X^2_1$ | *P-Value* |
|---|---|---|
| Sex (F *vs* M) | 0.07 | 0.79 |
| p53 ( + VE *vs* – VE) | 4.44 | 0.035 |
| EGFR ( + VE *vs* – VE) | 7.34 | 0.007 |
| Ki-67 LI (<5% *vs* >5%) | 20.50 | <0.0001 |
| Surgery (total *vs* subtotal *vs* partial *vs* biopsy) | $7.37 = X^2_3$[a] | 0.06 |
| Histopathology grade (1,2 *vs* 3,4) | 18.30 | <0.0001 |
| Radiotherapy ( + *vs* – ) | 0.62 | 0.43 |
| Chemotherapy ( + *vs* – ) | 0.01 | 0.94 |

[a]Note, 3 degrees of freedom.

**Figure 1** Survival curves for patients with astrocytomas grade 1 and 2 (–; $n = 9$) and patients with astrocytoma grade 3 and 4 (---; $n = 31$). Log-Rank statistic = 18.30; $P < 0.0001$; d.f. = 1.

endothelial proliferation. The nuclear labelling did not correlate with any particular tumour cell type. This was most obvious in such morphologically heterogeneous tumours such as glioblastoma multiforme or gigantocellular glioma where both small and large nuclei were either labelled or unlabelled (Figures 2 and 3). The intensity of the nuclear labelling within the tumour also varied: lesser or more intensely labelled nuclei were intermingled in an irregular fashion (Figures 2 and 3).

Tumour cell nuclei positively labelled with the p53 antibody were found in seven out of 16 types of tumours examined (Table IV, columns 1 and 2). In astrocytomas it should be noted that none of the grade 1 tumours were labelled but that the proportion of positive tumours increased with tumour grade (Table IV, column 3). P53 LI was variable within astrocytoma grades ranging from 1.2% to 29.4% in grade 3, and from 0.1% to 61.9% in grade 4 (Table I, column 6), and the mean LI was similar in the two grades ($P = 0.83$; Table V) indicating absence of correlation between p53 LI and the tumour grade, at least for the two malignant grades. In some tumours a striking regional heterogeneity in p53 + ve nuclei was evident in histological sections. For example, in a p53 + ve PNET (patient number 71) shown in Figure 4, p53 LI in two neighbouring areas was 0.1% and 21.8%. Several astrocytomas grade 3 and 4 also showed regional heterogeneity in the p53 labelling, the greatest differences in p53 LI was 1.2% and 22% in patient number 147 (Table I, column 6).

**Figure 2** Positive p53 mutant protein nuclear labelling in astrocytoma grade 4 (glioblastoma multiforme) with PAb 1801; both small and large nuclei were either labelled (brown) or unlabelled (blue). Scale bar = 30 μm.

**Figure 3** Positive p53 mutant protein nuclear labelling in gigantocellular glioma with PAb 1801; both small and large nuclei were either labelled (brown) or unlabelled (blue). Scale bar = 30 μm.

**Table IV** Expression of p53 and EGF receptor proteins in CNS tumours

| *1* | *2* | *3* | *4* | *5* |
|---|---|---|---|---|
| | *p53 + ve* | | *EGFR + ve* | |
| *Tumour type* | *Ratio* | *%* | *Ratio* | *%* |
| Astrocytoma Grade 1 | 0/4 | 0% | 0/4 | 0% |
| Grade 2 | 1/6 | 17% | 2/6 | 33% |
| Grade 3 | 5/13 | 38% | 11/13 | 85% |
| Grade 4[a] | 13/20 | 65% | 9/20 | 95% |
| Total | 19/43 | 44% | 32/43 | 74% |
| Primitive neuro-ectodermal tumours | 1/6 | 17% | 4/6 | 67% |
| Astroblastoma | 1/2 | | 2/2 | |
| Gigantocellular glioma | 1/1 | | 1/1 | |
| Oligodendroglioma | 0/1 | | 1/1 | |
| Ependymoma Grade 2/3 | 0/2 | | 2/2 | |
| Myxopapillary | 0/2 | | 0/2 | |
| Angioglioma | 0/3 | | 1/2 | |
| Choroid plexus papilloma | 0/3 | | 3/3 | |
| CNS dysgerminoma | 2/2 | | 1/2 | |
| Pituitary tumour | 0/3 | | 0/3 | |
| Haemangioblastoma | 0/3 | | 2/3 | |
| Chordoma | 0/1 | | 0/1 | |
| Tumours in Von Recklinghausen's | | | | |
| neurofibroma[b] | 0/1 | | 1/1 | |
| neurofibrosarcoma[c] | 1/1 | | 1/1 | |
| Angioma (developmental abnormality) | 0/1 | | 0/1 | |
| Metastatic tumours in CNS | | | | |
| carcinoma | 0/2 | | 0/1 | |
| melanoma | 1/1 | | 0/1 | |

[a]Includes glioblastoma multiforme. [b]Associated with spinal roots. [c]Intrinsic to cerebrum.

**Table V** p53, EGFR, and Ki-67 labelling in relation to histopathological tumour grade

| Grade | *p53 LI* *Mean (s.d.;n)* | *EGFR labelling factor* *Mean (s.d.;n)* | *Ki-67 LI* *Mean (s.d.;n)* |
|---|---|---|---|
| 1 & 2 | | 110.1(31.6;10) | 1.1(1.0;10) |
| | | *vs* 3 & 4: $P<0.0001$ | *vs* 3: $P = 0.01$ |
| | | d.f. = 41 | d.f. = 21 |
| 3 | 14.1 (12.2;13) | 247.6(113.1;13) | 8.3(9.1;13) |
| | *vs* 4: $P = 0.83$ | *vs* 4: $P = 0.1$ | *vs*4: $P = 0.006$ |
| | d.f. = 31 | d.f. = 31 | d.f. = 31 |
| 4 | 12.4(16.3;20) | 323.0(136.1;20) | 13.4(7.3;20) |
| 3 & 4 | | 293.1(131.2;33) | |

**Figure 4** Regional heterogeneity of positive p53 mutant protein nuclear labelling (brown) with PAb 1801 in primitive neuroectodermal tumour: in an area partly shown in the upper half of the figure p53 LI was 21.8%, in a neighbouring area, partly shown in the lower half of the figure, p53 LI was 0.1%. Ki-67 LI quantified from a serial section, was similar in the two areas, about 9%. Scale bar = 30 μm.

Patients with p53 + ve astrocytomas had a reduced survival ($P = 0.035$; Table III) – only 11% of these patients were alive at 100 weeks following operation and diagnosis compared to 36% of patients with p53-ve tumours (Figure 5). The number of cases with tumours in the other categories were too small to attempt survival analyses.

*Immunohistochemistry of EGFR*

No labelling was seen in normal neocortical (12 ×) and normal cerebellar (4 ×) nervous tissue adjacent to tumours or in tumour endothelial cells. The EGFR labelling of tumours was restricted to cytoplasmic regions and, in some instances, possibly to cell membranes of tumour cells in the EGFR + ve tumours (Figures 6 and 7). This is in contrast to normal human epidermis, which was used to determine the optimal dilution of the EGFR antibody, where the labelling was associated exclusively with cell membranes.

Eleven out of 16 types of tumours examined had EGFR positive cells (Table IV, column 1 and 4). It should be noted that a higher proportion of all astrocytomas was labelled with EGFR (74%) than p53 antibody but that, similar to p53 labelling, none of the grade 1 tumours were labelled, and the proportion of positive tumours increased with tumour grade (Table IV, column 5). In some tumours the EGFR labelling was intense and uniform both in terms of distribution and intensity (Figure 6) but in other tumours it was fainter (Figure 7) or patchy and of variable intensity (see also Table I, column 7). The variability of these two parameters was taken into account by calculating EGFR labelling factor for each tumour (see Material and methods). Astrocytomas grades 3 & 4 had a significantly higher mean labelling factor than grades 1 and 2 ($P<0.0001$; Table V) indicating that the intensity/area of EGFR labelling increased with malignancy grade. Other tumours with high degree of EGFR labelling included PNETs, astroblastomas, oligodendrogliomas, choroid plexus papillomas, and angiogliomas (Table I, column 7).

Patients with EGFR + ve astrocytomas appeared to have reduced survival ($P = 0.007$; Table III) – only 13% of these patients were alive at 100 weeks following diagnosis compared to 60% of EGFR-ve patients (Figure 8). The number of cases with tumours in the other categories were too small to attempt survival analysis.

**Figure 5** Survival curves for patients with p53 negative astrocytomas (p53neg–; $n = 22$) and patients with p53 positive astro cytomas (p53pos---; $n = 18$). Log-Rank statistic = 4.44; $P = 0.035$; d.f. = 1.

**Figure 6** Intense positive EGFR labelling (brown) in astrocytoma grade 4 (glioblastoma multiforme) with EGFR1 antibody; the blood vessel (v) is unlabelled. Scale bar = 30 $\mu$m.

**Figure 7** Faint EGFR labelling (pale brown) in gigantocellular glioma with EGFR1 antibody. Scale bar = 30 $\mu$m.

*Immunohistochemistry of Ki-67*

At the cellular level the Ki-67 antibody reactivity had an exclusively nuclear distribution, and was either uniform or granular (Figure 9). Most tumours had at least some Ki-67 labelled nuclei with the exception of one grade 2 astrocytoma in tuberous sclerosis, one angioglioma, one chordoma and and one angioma (Table I, column 8). Heterogeneity in Ki-67 nuclear labelling was found in around of 20% astrocytomas (Table I, column 8). However, the mean values of the Ki-67, LI, representing the proliferative potential of the tumour (see Material and methods), showed a statistically significant increase with increasing grade of astrocytoma malignancy (1 and 2 *vs* 3; $P = 0.01$; 3 *vs* 4: $P = 0.006$; Table V).

In the Univariate analysis the astrocytoma patients' Ki-67 LI, when analysed as a continuous variable by Cox regression analysis, showed a strong relationship to the length of surival ($P > 0.0001$; Table II). When analysed by the Log-Rank test patients with Ki-67 LI > 5% (5.1 to 30.9%) had a reduced survival ($P < 0.0001$; Table III) – none of these patients survived beyond 86 weeks following diagnosis compared with 63% of patients with Ki-67 LI of < 5% (0.1 to 3.9%) who were still alive at 100 weeks (Figure 10). The number of cases with tumours in the other categories were too small to attempt survival analysis.

**Figure 8** Survival curves for patients with EGF receptor negative astrocytomas (EGFRneg–; $n = 10$) and patients with EGF receptor positive astrocytomas (EGFRpos---; $n = 30$). Log-Rank statistic = 7.34; $P = 0.007$; d.f. = 1.

**Figure 9** Positive Ki-67 nuclear labelling (brown) in astrocytoma grade 4 (glioblastoma multiforme); a blood vessel (v) displays one labelled endothelial cell nucleus (arrow). Scale bar = 30 $\mu$m.

**Figure 10** Survival curves for patients with Ki-67 LI below 5% (<5%-; $n = 16$) and patients with Ki-67 LI above 5% (>5% ---; $n = 24$). Log-Rank statistic = 20.50; $P<0.0001$; d.f. = 1.

### *Relationship between p53, EGFR and Ki-67 labelling*

In about 25% of the astrocytomas regional heterogeneity was detected either in p53, EGFR, or Ki-67 labelling, and therefore more than one area was counted in those tumours (Table I, columns 6, 7, 8). Note that in most cases only one of the three antibodies showed heterogeneity, in a few cases two antibodies but never all three simultaneously. The relationship between p53, EGFR and Ki-67 labelling was determined in serial sections from corresponding areas but not between different areas. The p53 + ve astrocytomas had a significantly higher mean Ki-67 LI than p53-ve astrocytoma ($P = 0.036$; Table VI), although the values of the continuous variables, p53 LI and Ki-67 LI, did not show any correlation within the individual tumours ($r = 0.19$; $P = 0.17$). A pictorial example of absence of correlation should be noted in Figure 4 where regional heterogeneity in the p53 labelling cannot be explained by differences in proliferative activity between the two areas since they both had an almost identical Ki-67 LI of 9%.

The continuous variables, EGFR labelling factor and Ki-67 LI, were significantly correlated within the individual tumours ($r = 0.32$; $P = 0.018$). The EGFR + ve astrocytomas had a significantly higher mean Ki-67 LI than EGFR-ve astrocytomas ($P = 0.028$; Table VI). Astrocytomas which were both EGFR + ve and p53 + ve had a somewhat higher Ki-67 LI than astrocytomas which were EGFR + ve but p53-ve, though the difference was not significant ($P = 0.49$; Table VI). Astrocytomas not expressing either EGFR or p53 proteins had the lowest mean Ki-67 LI ($P<0.0001$; Table VI), although the continuous variables, p53 LI and EGFR labelling factor, showed no correlation within individual tumours ($r = 0.04$; $P = 0.86$). The absence of correlation is probably due to variability in p53 LI between tumours (see Table I, column 6), and also due to non-overlapping regional heterogeneity in EGFR and p53 labelling within individual tumours.

The effect of EGFR and p53 expression on patient's survival was not cumulative ($P = 0.66$; Figure 11) – similar proportions of patients with EGFR + ve & p53 + ve tumours (12%) were alive at 100 weeks following diagnosis compared to patients with EGFR + ve & p53-ve tumours (15%). In contrast, patients with EGFR-ve & p53-ve tumours had a significantly better survival rate than both of the previous groups (67%; $P = 0.016$; Figure 11).

### *Multivariate analysis*

The list of all variables which were analysed for prognostic importance by univariate analysis are shown in Table II (Cox's Regression for continuous variables) and in Table III (Log-Rank test for categorised variables). Note that the univariate prognostic importance of Ki-67 LI is of the same order of magnitude ($P<0.0001$) whether it is considered as a

**Figure 11** Survival curves for patients with p53 negative and EGFR negative astrocytomas (EGFRneg and p53pos–; $n = 9$), patients with p53 negative and EGFR positive astrocytomas (EGFRpos and p53neg ---; $n = 13$), and patients with p53 positive and EGFR positive astrocytomas (EGFRpos and p53pos . . .; $n = 17$). Log-Rank statistic = 8.29; $P = 0.016$; d.f. = 2. The only patient with a p53 positive and EGFR negative astrocytoma died 24 weeks after diagnosis. For EGFRpos and p53neg *vs* EGFRpos & p53pos survival curves the Log-Rank statistic = 0.19; $P = 0.66$; d.f. = 1.

**Table VI** Ki-67 labelling in relation to p53 and EGFR labelling

| | *Ki-67 LI Mean (SD;n)* | *P-value* | *Degrees of freedom* |
|---|---|---|---|
| p53-ve | 6.2(6.6;24) | | |
| | *vs* | 0.036 | 41 |
| p53 + ve | 11.8(10.2;19) | | |
| EGFR-ve | 4.1(6.3;11) | | |
| | *vs* | 0.028 | 41 |
| EGFR + ve | 10.7(8.8;32) | | |
| p53-ve & EGFR + ve | 9.0(7.3;14) | | |
| | *vs* | 0.49 | 30 |
| p53 + ve & EGFR + ve | 11.2(10.2;18) | | |
| p53-ve & EGFR-ve | 2.3(2.7;10) | | |
| | *vs* | <0.0001 | 40 |
| EGFR + ve (= p53-ve & EGFR + ve plus p53 + ve & EGFR + ve) | 10.7(8.8;32) | | |
| p53 + ve & EGFR-ve | 21.6 (1) | | |

continuous or categorical (<5% *vs* >5%) variable. Sex, radiotherapy and chemotherapy had non-significant univariate *P*-values, while surgery was of weak statistical importance ($P = 0.06$; Table III). All variables apart from sex, radiotherapy and chemotherapy were entered into a multivariate analysis to determine whether they influence the patient's prognosis independently or are associated with each other. The multivariate analysis was performed by using a forward stepwise application of Cox's Regression model. Tables VII and VIII shows that the only variables selected as statistically significant ($P < 0.10$) by this procedure were histopathological grade and Ki-67 LI. Although several of the variables had a significant prognostic importance following univariate analysis (Tables II and III), the multivariate analysis reveals histopathological grade as the overwhelming dominating factor with Ki-67 LI being the only other variable with prognostic information once histopathological grade has entered the model. By employing the regression procedure with only Ki-67, EGFR and p53 labelling as independent variables, only Ki-67 labelling was significant ($P = 0.0002$), and therefore the controlling variable. This is because many of the variables do not influence the survival of astrocytoma patients independently, and are interrelated with each other as follows, histopathological grade and age: 1 and 2 *vs* 3 and 4; $P < 0.0001$; histopathological grade and Ki-67 LI: 1 and 2 *vs* 3: $P = 0.01$, 3 *vs* 4; $P = 0.006$; histopathological grade and EGFR labelling factor: 1 and 2 *vs* 3 and 4: $P < 0.0001$; age and Ki-67 LI: $r = 0.37$, $P = 0.014$; age and EGFR labelling factor: $r = 0.57$, $P < 0.0001$; Ki-67 LI and EGFR labelling factor: $r = 0.32$, $P = 0.018$; except for histopathological grade and p53 LI: $P = 0.83$; age and p53 LI: $r = 0.06$, $P = 0.77$; Ki-67 LI and p53 LI: $r = 0.19$, $P = 0.17$; and EGFR labelling factor and p53 LI: $r = 0.04$, $P = 0.86$.

### Discussion

This study demonstrates immunohistochemically detectable levels of p53 protein in tumour cell nuclei of many CNS and non-CNS tumours. The PAb 1801 monoclonal antibody used in this study can recognize both normal and mutant forms of human p53 proteins (Banks *et al.*, 1986; Rodrigues *et al.*, 1990) but the labelling almost certainly represents accumulation of nonfunctional p53 mutants only. The mutants are detectable by immunohistochemistry (Cattoretti *et al.*, 1988; Iggo *et al.*, 1990; Rodrigues *et al.*, 1990) because of their metabolic stability, and their cellular levels are elevated 10–100 fold above normal values (Finlay *et al.*, 1989). In the present series none of the normal CNS tissue adjacent to the tumours was labelled with PAb 1801 antibody. Also, the labelling of tumour cells is unlikely to represent the somewhat elevated levels of normal p53 seen in actively proliferating cell populations (Dippold *et al.*, 1981; Levin & Momand, 1990) because some tumours with large growth fractions were not labelled with the PAb 1801 antibody, whilst in p53 + ve tumours the p53 LI did not correlate with the growth fraction size. In addition, no endothelial cells were labelled in any of the tumours, even in areas where endothelial proliferation, and Ki-67 labelling were present.

Our finding that p53 mutants are expressed in astrocytomas, primitive neuroectodermal tumour, astroblastomas, gigantocellular glioma, neurofibrosarcoma, CNS dysgerminomas, and melanoma extends the number of human tumours with identified mutations in p53 gene. So far, the list included carcinomas of the breast, lung, colorectum, and liver, and also neurofibrosarcoma, osteosarcoma and glioblastomas multiforme (Masuda *et al.*, 1987; Cattoretti *et al.*, 1988; Nigro *et al.*, 1989; Iggo *et al.*, 1990; Rodrigues *et al.*, 1990; Menon *et al.*, 1990; Bressac *et al.*, 1991; Hsu *et al.*, 1991). Using karyotyping we have not detected abnormalities of chromosome 17p (where gene p53 is localised) in any of the p53 + ve tumours in this study. This may not be surprising since accumulation of p53 protein is almost certainly an outcome of point mutations in the p53 gene (Nigro *et al.*, 1989) which is beyond resolution of karyotyping.

In the present series the p53 labelling was exclusively localised in tumour cell nuclei, whilst in other tumour types either nuclear or a combined nuclear and cytoplasmic p53 labelling has been found (Iggo *et al.*, 1990; Rodrigues *et al.*, 1990). The nuclear localisation may indicate presence of transforming p53 mutants. Normal p53 protein is thought to have a role in regulating gene expression or DNA replication (Michalovitz *et al.*, 1991), and it appears that nuclear localisation of p53 mutants is essential for their transforming activity (Shaulsky *et al.*, 1990). The reason for some p53 mutants accumulating in the nuclei is unclear. Cytoplasmic accumulation of some p53 mutants has been reported to occur because a conformational change in their molecule leads them to form complexes with cytoplasmic heat-shock-cognate protein 70 (hsc 70; Sturtzbecher *et al.*, 1988). It would be of interest to see whether the p53 mutants that accumulate in nuclei of different tumour types in this and other studies share a particular conformational change, and bind to an as yet unidentified nuclear protein.

In astrocytomas this study has found that the nuclear

**Table VII** Variables that achieved $P < 0.10$ following forward stepwise Cox regression on all variables, Ki-67 LI analysed as continuous variable

| *Variable* | *Coefficient* | *SE* | *Relative risk (95% C.I.)*[a] | *P-value* | $\chi^2_1$ *to enter model* |
|---|---|---|---|---|---|
| Histological grade | 2.920 | 1.050 | 18.50 (2.37,145.2) | 0.005 | 26.23 |
| Ki-67 LI | 0.044 | 0.025 | 1.045 (0.99,1.10) | 0.08 | 2.99 |

[a]95% C.I. = 95% Confidence Interval for Relative Risk is given by $e^{\text{coefficient} \pm 1.96\text{SE(coefficient)}}$.

**Table VIII** Variables that achieved $P < 0.10$ following forward stepwise Cox regression on all variables, Ki-67 LI categorical <5% *vs*>5%

| *Variable* | *Coefficient* | *SE* | *Relative risk (95% C.I.)*[a] | *P-value* | $\chi^2_1$ *to enter model* |
|---|---|---|---|---|---|
| Histological grade | 2.694 | 1.082 | 14.80 (1.78,123.2) | 0.012 | 26.23 |
| Ki-67 LI | 0.975 | 0.508 | 1.921 (0.98,7.17) | 0.055 | 4.31 |

[a]95% C.I. = 95% Confidence Interval for Relative Risk is given by $e^{\text{coefficient} \pm 1.96\text{SE(coefficient)}}$.

expression of p53 mutants was associated with increase in tumour malignancy and poor prognosis. Using immunohistochemistry, similar observations, though without survival data, have been made in neurofibrosarcoma, and carcinomas of the breast, lung, and colorectum (Baker *et al.*, 1989; Vogelstein *et al.*, 1989; Iggo *et al.*, 1990; Harris *et al.*, 1990; Menon *et al.*, 1990). In astrocytomas p53 expression has not yet been examined by immunohistochemistry but allele loss of chromosome 17p, and presumed mutations in p53 gene, have been reported to be associated with tumour initiation (James *et al.*, 1989; El-Azouzi *et al.*, 1989) which contrasts with the findings of the present study. The reason for this apparent difference between conclusions of the present and previous studies may be due to the difference in sensitivities of the different techniques employed, and may not emerge until the mechanism of tumour evolution in astrocytomas is more precisely understood at molecular level. However, several possible explanations might be considered. Firstly, low and high grade astrocytomas may originate from different tumour precursor cells (James *et al.*, 1988). The precursor cells giving rise to low grade astrocytomas may be affected by loss-of-function p53 mutations leading to a failure to express any p53 RNA and protein, similar to that reported in a proportion of rhabdomyosarcomas, osteosarcomas, and Li-Fraumeni lesions (Masuda *et al.*, 1987; Mulligan *et al.*, 1990; Malkin *et al.*, 1990). Only precursors giving rise to high grade astrocytomas may be affected by transforming p53 mutations leading to overexpression of the p53 mutants, similar to that reported in carcinoma of the breast and lung (Cattoretti *et al.*, 1988; Iggo *et al.*, 1990). This scheme, however, implies that benign astrocytomas cannot progress to a malignant stage, which is contrary to clinical and histopathological observations (Russell & Rubinstein, 1989). Alternatively, if astrocytomas progress from low to higher grades (James *et al.*, 1988), they may do so by step-wise changes in the p53 gene, analogous to those proposed for colorectal carcinomas: the first or initiating step involving mutation in one allele only and a synthesis of inactive mutant/normal oligomers; further loss of control is believed to result from deletion of the normal allele, leaving the cell with only a mutant allele (Nigro *et al.*, 1989). The present immunohistochemical essay has detected p53 mutant molecules, but may not have been sufficiently sensitive to detect cells expressing oligomeric p53 mutant/normal molecules, similar to observation made by Rodrigues *et al.* (1990) on cell lines derived from acute lymphoblastic leukaemia. The finding that p53 LI was variably expressed between tumours, and regionally heterogeneous within tumours, indicates that p53 mutant molecules were expressed in subclones of astrocytic tumour cells, which were not present at the initial stages of tumour development. If a precursor cell expressed p53 mutants, all the daughter tumour cells in high grade astrocytomas would also express the mutant molecules.

In this series EGFR was expressed in astrocytomas and ten other tumour types, mostly with glial and/or neuroepithelial differentiation, but not in normal brain tissue adjacent to the tumours. In astrocytomas EGFR expression was associated with increase in tumour malignancy and poor prognosis. Our data confirm findings of previous biochemical (Liberman *et al.*, 1984; 1985) and immunohistochemical studies on astrocytomas (Reifenberger *et al.*, 1989), and extend them by survival data. But, unlike human breast cancer (Harris *et al.*, 1990), this series does not support a direct association between expression of EGFR and p53 mutants: there was no correlation between p53 LI and categories of EGFR labelling, possibly due to nonoverlapping regional heterogeneity in each parameter. This suggests that in astrocytomas p53 mutants and EGFR are expressed in different subclones of tumour cells, and that the associations between increase in tumour malignancy and the expression of EGFR protein or p53 mutants occur independently of each other.

Overexpression of EGFR, previously found to occur in a high proportion of malignant gliomas, has been related either to an amplification of the EGFR gene, often in the form of double minutes (Liberman *et al.*, 1984; 1985; Wong *et al.*, 1987), or to an extra copy of chromosome 7 (Liberman *et al.*, 1984) on which the EGFR gene is located (Shimizu *et al.*, 1985), or to loss of control of transcriptional activity of the gene (Gerosa *et al.*, 1989). In this study trisomy of chromosome 7 was found only in one patient, and double minutes in none. It therefore seems that in the present series the possible mechanisms responsible for EGFR overexpression are either amplification at the EGFR gene locus which is not easily detectable by karyotyping, or loss of control of transcriptional activity. Our observation that in the majority of the brain tumours EGFR labelling had a predominantly cytoplasmic distribution may be explained by rapid internalisation of the EGR after ligand binding (Stoscheck & King, 1986; Humphrey *et al.*, 1990). Alternatively, similar to human glioma cell lines which show co-expression of high levels of EGFR and one of its ligands TGF-$\alpha$ (Nister *et al.*, 1988), an autocrine growth stimulation loop may operate in astrocytomas *in vivo*, and the cytoplasmic labelling may represent cytoplasmic binding of EGFR to its ligand.

In this series high Ki-67 LI was associated with reduced survival. This is contrary to the only previous study which included survival data (Zuber *et al.*, 1988), possibly due to a small sample size in the previous study. It is likely that the poor prognosis found in patients with Ki-67 LI higher than 5% reflects a significant correlation between mean size of the growth fraction, as determined with the Ki-67 index, and histopathological grade of malignancy in astrocytomas. The latter observation is in broad agreement with previous studies (Raghavan *et al.*, 1990; Brown & Gatter, 1990) but the conclusions of the present study go further – the Multivariate analysis has demonstrated that the histopathological grade is the most important variable to influence the patient's survival, when all variables are considered together. The analysis has also demonstrated that histopathological grade, age, Ki-67 LI, and EGFR and p53 labelling do not influence survival of astrocytoma patients independently but, except for the p53 labelling, are interrelated with each other. The proliferative ability of astrocytoma cells, as determined by Ki-67 LI, appeared to be positively influenced by expression of both p53 mutants and EGFR protein, since p53 + ve or EGFR + ve astrocytomas had significantly higher Ki-67 indices than p53-ve or EGFR-ve astrocytomas but the Ki-67 LI is the most important variable to influence the patient's survival when considered together with the EGFR and p53 labelling. This finding and two other observations indicate that additional or alternative mechanisms to expression of p53 mutants and EGFR are likely to be involved in controlling tumour cell proliferation and tumour progression in astrocytomas. Firstly, in p53-ve and/or EGFR-ve astrocytomas tumour cells also displayed proliferative activity, even though it was lower than in p53 + ve and/or EGFR + ve tumours. Secondly, though EGFR labelling correlated positively with Ki-67 LI, analysis of p53 LI and Ki-67 LI did not show a significant correlation. These conclusions are in keeping with earlier cytogenetic findings which have implicated multiple chromosomal abnormalities in malignant progression of gliomas in addition to 17p and 7, where p53 and EGFR genes respectively, are localised (Bigner *et al.*, 1984; Shapiro, 1986; James *et al.*, 1988). In model for progression of gliomas Bigner and Vogelstein (1990) proposed that abnormality on chromosome 17p occurs at early stages of tumorigenesis, whilst EGFR is thought to stimulate further progression of malignant gliomas. This study has demonstrated that in astrocytomas expression of both p53 mutants and EGFR can occur at early stages of tumorigenesis, and that their expression also represents mechanisms associated with malignant progression and poor prognosis but that expression of neither protein may be essential for this process.

The support of the North of England Cancer Research Campaign and of the North of England Children's Cancer Research Fund is gratefully acknowledged. The survival curves were generated and analysed by Logrank statistics using a programme developed by J. Smith and M. Cole in the Department of Child Health, the University of Newcastle upon Tyne. Thanks are due to Billy McMeekin for technical advice and to Mrs S. Hammond for typing the manuscript.

### References

ARITA, N., HAYAKAWA, T., IZUMOTO, S. & 5 others (1989). Epidermal growth factor receptor in human glioma. *J. Neurosurg.*, **70**, 916.

BAKER, S.J., FEARON, E.R., NIGRO, J.M. & 9 others (1989). Chromosome 17 deletions and p53 gene mutations in colorectal carcinomas. *Science*, **244**, 217.

BANKS, L., MATLASHEWSKI, G. & CRAWFORD, L. (1986). Isolation of human-p53-specific monoclonal antibodies and their use in the studies of human p53 expression. *Eur. J. Biochem.*, **159**, 529.

BIGNER, S.H., MARK, J., FRIEDMAN, H.S., BIEGEL, J.A. & BIGNER, D.D. (1988). Structural abnormalities in human medulloblastoma. *Cancer Genet. Cytogenet.*, **30**, 91.

BIGNER, S.H., MARK, J., MAHALEY, Jr. M.S. & BIGNER, D.D. (1984). Patterns of the early gross chromosomal changes in malignant human gliomas. *Hereditas*, **101**, 103.

BIGNER, S.H. & VOGELSTEIN, B. (1990). Cytogenetics and molecular genetics of malignant gliomas and medulloblastomas. *Brain Pathol.*, **1**, 12.

BRESSAC, B., KEW, M., WANDS, J. & OZTURK, M. (1991). Selective G to T mutations of p53 gene in hepatocellular carcinoma from southern Africa. *Nature*, **350**, 429.

BROWN, D.C. & GATTER, K.C. (1990). Monoclonal antibody Ki-67: its use in histopathology. *Histopathology*, **17**, 489.

CARPENTER, G. (1987). Receptors for epidermal growth factor and other polypeptide milogens. *Annu. Rev. Biochem.*, **56**, 881.

CATTORETTI, G., RILKE, F., ANDREOLA, S., D'AMATO, L. & DELIA, D. (1988). P53 expression in breast cancer. *Int. J. Cancer*, **41**, 178.

COX, D.R. (1972). Regression models and lifetables. *J. R. Stat. Soc.*, **34**, 187.

DIPPOLD, W.G., JAY, G., DELEO, A.B., KHOURY, G. & OLD, L.J. (1981). P53 transformation-related protein: detection by monoclonal antibody in mouse and human cells. *Proc. Natl Acad. Sci. USA*, **78**, 1695.

EL-AZOUZI, M., CHUNG, R.Y., FARMER, G.E. & 10 others (1989). Loss of distinct regions on the short arm of chromosome 17 associated with tumorigenesis of human astrocytomas. *Proc. Natl Acad. Sci. USA*, **86**, 7186.

FINLAY, C.A., HINDS, P.W. & LEVINE, A.J. (1989). The p53 protooncogene can act as a suppressor of transformation. *Cell*, **57**, 1083.

GEROSA, M.A., TALARICO, D., FOGNANI, C. & 5 others (1989). Overexpression of N-ras oncogene and epidermal growth factor receptor gene in human glioblastomas. *J. Natl Cancer Inst.*, **81**, 63.

GERDES, J., LEMKE, H., BAISCH, H., WACKER, H.H., SCHWAB, U. & STEIN, H. (1984). Cell cycle analysis of a proliferation associated human nuclear antigen defined by the monoclonal antibody Ki-67. *J. Immunol.*, **133**, 1710.

GRIFFIN, C.A., HAWKINS, A.L., PACKER, R.J., RORKE, L.B. & EMANUEL, B.S. (1988). Chromosome abnormalities in paediatric tumours. *Cancer Res.*, **48**, 175.

HARRIS, A.L., HORAK, E., SMITH, K. & 4 others (1990). Mutant p53 is a common genetic abnormality in human breast cancer and associated with EGF receptor and neu expression. BACR 31st Meeting. *Brit. J. Cancer*, **62**, 503 (Abstr.).

HSU, I.C., METCALF, R.A., SUN, T., WELSH, J.A., WANG, N.J. & HARRIS, C.C. (1991). Mutational hotspot in the p53 gene in human hepatocellular carcinomas. *Nature*, **350**, 427.

HUMPHREY, P.A., WONG, A.J., VOGELSTEIN, B. & 7 others (1990). Anti-synthetic peptide antibody reacting at the fusion junction of deletion-mutant epidermal growth factor receptors in human glioblastoma. *Proc. Natl. Acad. Sci. USA*, **87**, 4207.

HUNTER, T. (1984). The epidermal growth factor receptor gene and its product. *Nature*, **311**, 414.

IGGO, R., GATTER, K., BARTEK, J., LANE, D. & HARRIS, A.L. (1990). Increased expression of mutant forms of p53 oncogene in primary lung cancer. *Lancet*, **335**, 675.

JAMES, C.D., CARLBOM, E., DUMANSKI, J.P. & 4 others (1988). Clonal genomic alterations in glioma malignancy stages. *Cancer Res.*, **48**, 5546.

JAMES, C.D., CARLBOM, E., NORDENSKJOLD, M., COLLINS, V.P. & CAVENEE, W.K. (1989). Mitotic recombination of chromosome 17 in astrocytomas. *Proc. Natl Acad. Sci. USA*, **86**, 2858.

JAROS, E., PEARSON, A.D.J. & PERRY, R.H. (1991*b*). Immunohistochemical study of p53 protein, epidermal growth factor receptor (EGFR) and Ki-67 labelling in brain tumours. 82nd meeting of British Neuropath Society. *Neuropath. Appl. Neurobiol.*, **17**, 522 (Abstract).

JAROS, E., PERRY, R.H., PEARSON, A.D.J. & 4 others (1991*a*). p53 expression in brain tumours. 10th Meeting of the British Neurooncology Group. *Brit. J. Neurosurg.*, **5**, 211 (Abstract).

KERNOHAN, J.W., MABON, R.F., SVIEN, H.J. & ADSON, A.W. (1949). Symposium on a new and simplified concept of gliomas. A simplified classification of gliomas. *Proc. Mayo Clinic*, **24**, 71.

LEVINE, A.J. & MOMAND, J. (1990). Tumour suppressor genes: the p53 and retinoblastoma sensitivity genes and gene products. *Biochim. Biophys. Acta*, **1032**, 119.

LIBERMANN, T.A., NUSBAUM, H.R., RAZON, N. & 7 others (1985). Amplification, enhanced expression and possible rearrangements of EGF receptor gene in primary human brain tumours of glial origin. *Nature*, **313**, 144.

LIBERMANN, T.A., RAZON, N., BARTAL, A.D., YARDEN, Y., SCHLESSIGER, J. & SOREQ, H. (1984). Expression of Epidermal Growth Factor Receptors in human brain tumours. *Cancer Res.*, **44**, 753.

MALKIN, D., LI, F.P., STRONG, L.C. & 8 others (1990). Germ line mutations in familial syndrome of breast cancer, sarcomas, and other neoplasms. *Science*, **250**, 1233.

MASUDA, H., MILLER, C., KOEFFLER, H.P., BATTIFORA, H. & CLINE, M.J. (1987). Rearrangement of the p53 gene in human osteogenic sarcomas. *Proc. Natl Acad. Sci. USA*, **84**, 7716.

MENON, A.G., ANDERSON, K.M., RICCARDI, V.M. & 13 others (1990). Chromosome 17p deletions and p53 gene mutations associated with the formation of malignant neurofibrosarcomas in von Recklinghausen neurofibromatosis. *Proc. Natl Acad. Sci. USA*, **87**, 5435.

MICHALOVITZ, D., HALEVY, O. & OREN, M. (1991). p53 mutations: gains or losses? *J. Cell. Biochem.*, **45**, 22.

MULLIGAN, L.M., MATLASHEWSKI, G.J., SCRABLE, H.J. & CAVENEE, W. (1990). Mechanisms of p53 loss in human carcinomas. *Proc. Natl Acad. Sci. USA*, **87**, 5863.

NISTER, M., LIBERMANN, T.A., BETSHOLTZ, C. & 5 others (1988). Expression of messenger RNAs for platelet-derived growth factor and transforming growth factor-α and their receptors in human malignant cell lines. *Cancer Res.*, **48**, 3910.

NIGRO, J.M., BAKER, S.J., PREISINGER, A.C. & 13 others (1989). Mutations in the p53 gene occur in diverse human tumour types. *Nature*, **342**, 705.

PETO, R., PIKE, M.C., ARMITAGE, P. & 7 others (1977). Design and analysis of randomized clinical trials requiring prolonged observation of each patient. II. Analysis and examples. *Br. J. Cancer*, **35**, 1.

RAGHAVAN, R., STEART, P.V. & WELLER, R.O. (1990). Cell proliferation patterns in the diagnosis of astrocytomas, anaplastic astrocytoma and glioblastoma multiforme: a Ki-67 study. *Neuropathol. Appl. Neurobiol.*, **16**, 123.

REIFENBERGER, G., PRIOR, R., DECKERT, M. & WECHSLER, W. (1989). Epidermal growth factor receptor expression and growth fraction in human tumours of the nervous system. *Virchows Archiv A Pathol. Anat.*, **414**, 147.

RODRIGUES, N.R., ROWAN, A., SMITH, M.E.F. & 4 others (1990). p53 mutations in colorectal cancer. *Proc. Natl Acad. Sci. USA*, **87**, 7555.

RORKE, L.B., GILES, F.H., DAVIS, R.L. & BECKER, L.E. (1985). Revision of the world health organization classification of brain tumours for childhood brain tumours. *Cancer*, **56**, 1869.

RUSSELL, D.S. & RUBINSTEIN, L.J. (1989). *Pathology of Tumours of the Nervous System.* 5th ed. Edward Arnold. A division of Hodder and Stoughton: London, Melbourne, Auckland.

SHAPIRO, J.R. (1986). Biology of gliomas: heterogeneity, oncogenes, growth factors. *Semin. Oncol.*, **13**, 4.

SHAULSKY, G., GOLDFINGER, N., BEN-ZE'EV, A. & ROTTER, V. (1990). Nuclear accumulation of p53 protein is mediated by several nuclear localization signals and plays a role in tumourigenesis. *Mol. Cell. Biol.*, **10**, 6565.

SHIMIZU, N., HUNTS, J., MERLINO, G. & 5 others (1985). Regional mapping of the EGF receptor (EGFR/c-erbB) proto-oncogene. *Cytogenet. Cell Genet.*, **40**, 743.

STANBRIDGE, E.J. (1990). Human tumour suppressor genes. *Annu. Rev. Genet.*, **24**, 615.

STOSCHECK, C.M. & KING, Jr. L.E. (1986). Functional and structural characteristics of EGF and its receptor and their relationship to transforming proteins. *J. Cell. Biochem.*, **31**, 135.

STURZBECHER, H.-W., ADDISON, C. & JENKINS, J.R. (1988). Characterization of mutant p53-hsp72/73 protein-protein complexes by transient expression in monkey COS cells. *Mol. Cell. Biol.*, **8**, 3740.

SUGAWA, N., EKSTRAND, A.J., JAMES, C.D. & COLLINS, V.P. (1990). Identical splicing of aberrant epidermal growth factor receptor transcripts from amplified rearranged genes in human glioblastomas. *Proc. Natl Acad. Sci. USA*, **87**, 8602.

THOMAS, G.A. & RAFFEL, C. (1991). Loss of heterozygosity on 6q, 16q, and 17p in human central nervous system proimitive neuroectodermal tumours. *Cancer Res.*, **51**, 639.

VOGELSTEIN, B., FEARON, E.R., BAKER, S.J. & 7 others (1989). Genetic alterations accumulate during colorectal tumorigenesis. In *Recessive Oncogenes and Tumour Suppression*. Cavenee, W., Hastie, N. & Stanbridge (eds) p. 73. Cold Spring Harbor Laboratory Press.

WONG, A.J., BIGNER, S.H., BIGNER, D.D., KINZLER, K.W., HAMILTON, S.R. & VOGELSTEIN, B.E. (1987). Increased expression of the epidermal growth factor receptor gene in malignant gliomas is invariably associated with gene amplification. *Proc. Natl Acad. Sci. USA*, **84**, 6899.

ZUBER, P., HAMOU, M.-F. & DE TRIBOLET, N. (1988). Identification of proliferating cells in human gliomas using the monoclonal antibody Ki-67. *Neurosurgery*, **22**, 364.